# Protein Crystals in Adenovirus Type 5-Infected Cells: Requirements for Intranuclear Crystallogenesis, Structural and Functional Analysis

**DOI:** 10.1371/journal.pone.0002894

**Published:** 2008-08-06

**Authors:** Laure Franqueville, Petra Henning, Maria Magnusson, Emmanuelle Vigne, Guy Schoehn, Maria E. Blair-Zajdel, Nagy Habib, Leif Lindholm, G. Eric Blair, Saw See Hong, Pierre Boulanger

**Affiliations:** 1 Université Lyon I, Faculté de Médecine Laënnec, Laboratoire de Virologie et Pathologie Humaine, CNRS-FRE-3011, Lyon, France; 2 Institute for Biomedicine, Department of Microbiology and Immunology, University of Göteborg, Göteborg, Sweden; 3 Got-A-Gene AB, Östra Kyviksvägen 18, Kullavik, Sweden; 4 Sanofi-Avantis, Centre de Recherches de Vitry, Vitry-sur-Seine, France; 5 Université de Grenoble Joseph Fourier (UJF), Unit for Virus-Host Cell Interactions, UMR-5233 UJF-EMBL-CNRS, and Institut de Biologie Structurale Jean-Pierre Ebel, UMR-5075 CEA-CNRS-UJF, Grenoble, France; 6 Biomedical Research Centre, Sheffield Hallam University, Sheffield, United Kingdom; 7 Department of Surgical Oncology and Technology, Imperial College, Hammersmith Hospital Campus, London, United Kingdom; 8 Institute of Molecular and Cellular Biology, Faculty of Biological Sciences, University of Leeds, Leeds, United Kingdom; 9 Laboratoire de Virologie Médicale, Centre de Biologie et Pathologie Est, Hospices Civils de Lyon, Bron, France; Institut Pasteur Korea, Republic of Korea

## Abstract

Intranuclear crystalline inclusions have been observed in the nucleus of epithelial cells infected with Adenovirus serotype 5 (Ad5) at late steps of the virus life cycle. Using immuno-electron microscopy and confocal microscopy of cells infected with various Ad5 recombinants modified in their penton base or fiber domains, we found that these inclusions represented crystals of penton capsomers, the heteromeric capsid protein formed of penton base and fiber subunits. The occurrence of protein crystals within the nucleus of infected cells required the integrity of the fiber knob and part of the shaft domain. In the knob domain, the region overlapping residues 489–492 in the FG loop was found to be essential for crystal formation. In the shaft, a large deletion of repeats 4 to 16 had no detrimental effect on crystal inclusions, whereas deletion of repeats 8 to 21 abolished crystal formation without altering the level of fiber protein expression. This suggested a crucial role of the five penultimate repeats in the crystallisation process. Chimeric pentons made of Ad5 penton base and fiber domains from different serotypes were analyzed with respect to crystal formation. No crystal was found when fiber consisted of shaft (S) from Ad5 and knob (K) from Ad3 (heterotypic S5-K3 fiber), but occurred with homotypic S3K3 fiber. However, less regular crystals were observed with homotypic S35-K35 fiber. TB5, a monoclonal antibody directed against the Ad5 fiber knob was found by immunofluorescence microscopy to react with high efficiency with the intranuclear protein crystals *in situ*. Data obtained with Ad fiber mutants indicated that the absence of crystalline inclusions correlated with a lower infectivity and/or lower yields of virus progeny, suggesting that the protein crystals might be involved in virion assembly. Thus, we propose that TB5 staining of Ad-infected 293 cells can be used as a prognostic assay for the viability and productivity of fiber-modified Ad5 vectors.

## Introduction

In order to redirect adenovirus (Ad) virions to desired cell targets and transform them into cell-specific vectors suitable for biotherapy, diverse modifications of the adenoviral capsid have been designed and tested experimentally in various laboratories. These modifications have mainly concerned the projecting capsomer referred to as the fiber, and more particularly its distal globular domain (called the ‘knob’) involved in cell receptor recognition and attachment (reviewed in [1]). However, the fiber knob domain carries one of the signals required for trimerisation of Ad2 and Ad5 fibers [2]–[5], and modifications of this domain could have deleterious effects on the fiber structure and functions. Other important functions at the early phase of the virus life cycle have been assigned to the knob in addition to cell attachment, e.g. a role in intracellular trafficking [6]–[10]. At the late phase of the virus life cycle, the fiber knob-CAR interaction is considered as being responsible for the disruption of the tight junctions between epithelial cells [11]. The shaft domain, which governs the fiber length and flexibility, has been shown to be essential for efficient virus entry via the CAR-integrin pathway [12], [13]. Furthermore in the absence of available or accessible CAR molecules, Ad5 fiber shaft has been considered as the capsid component involved in cellular attachment through the interaction of the KKTK motif in its third repeat with cellular heparan sulfate proteoglycans [14].

In earlier constructions of Ad5 vectors with modified cellular tropism, we have observed that the deletion of the knob domain, compensated for by the insertion of a nonviral trimerisation motif, had deleterious effects on infectivity, growth rate and even viability of the modified viruses. This occurred despite the trimeric status of the fibers, their normal *O*-glycosylation and nuclear import, and the insertion of proper cell ligands for their binding to and propagation in receptor-displaying host cells [15]–[17]. Further investigation revealed that the fiber content of knob-deleted virions was lower than the theoretical number of 12 copies per virus particle, and reinsertion of the knob domain into the same fiber construct restored the fiber content to normal or subnormal copy number [15]–[17]. We recently showed that the low fiber content of the knob-deleted virions paralleled the lower fiber content of the virus-infected cells, compared to Ad5WTFib-infected cells, and we identified the translation of the knobless fiber mRNA as the step of the cell biosynthesis machinery which was altered upon knob deletion [18]. Our results therefore indicated that the knob domain plays a key role in the fiber content of the virion. Furthermore, results from other studies showed that Ad fiber is involved in the process of virus assembly and maturation : Ad5 mutants with fiber deletion or substitutions produce more empty capsids, have impaired maturation and decreased infectivity, compared to AdWTFib [7], [19], [20].

Since the nucleoplasm of Ad-infected cells is the compartment of virion assembly [1], we investigated the Ad5 assembly process *in situ*, using electron microscopy (EM), immuno-EM and immunofluorescence (IF) microscopy of cells infected with Ad5WT and a panel of Ad vectors with genetic modifications in the fiber or penton base genes. We observed profound alterations in the pattern of intranuclear assembly of virions and viral protein inclusions in cells infected with fiber deletion mutants : a number of particles lacked their electron-dense core and the intranuclear crystalline inclusions of viral proteins visible in Ad5WT-infected cells were absent or abnormal in structure. Intranuclear protein crystals have been already described [21]–[28]. Their structural parameters have been determined in Ad2-infected KB cells [25], but their exact nature and function, if any, have not been elucidated.

In the present study, we showed that the Ad-induced intranuclear protein inclusions represented crystals of penton capsomers, a hetero-oligomeric protein formed of a penton base and a fiber moiety. Analysis of Ad5 penton base and fiber mutants indicated that the accessible penton base RGD motifs were not involved in crystal formation. In Ad5 fiber however, the shaft domain comprising of repeats 17–21 and amino acid residues 489–492 within the knob domain were found to be essential for crystal formation. Interestingly, the absence of crystalline inclusions in certain Ad5-infected cells was associated with a lower fiber content and lower infectivity of the virus progeny, suggesting that the Ad5-induced intranuclear protein inclusions might serve as a privileged platform for efficient fiber encapsidation, correct capsid assembly, virus maturation and infectivity. A monoclonal antibody (mAb) which was directed against the Ad5 fiber knob (referred to as TB5) and cross-reacted with Ad2 and Ad3 knob but not with Ad35, was found by IF microscopy to react with high efficiency with the intranuclear protein crystals *in situ*. Thus, the IF pattern of Ad5-infected 293 cells with TB5 could be used as a rapid screening approach to assess viability and virion infectivity of recombinant Ad vectors.

## Results

### Intranuclear assembly pattern of Ad5 vector with WT fibers

**Figure 1:**
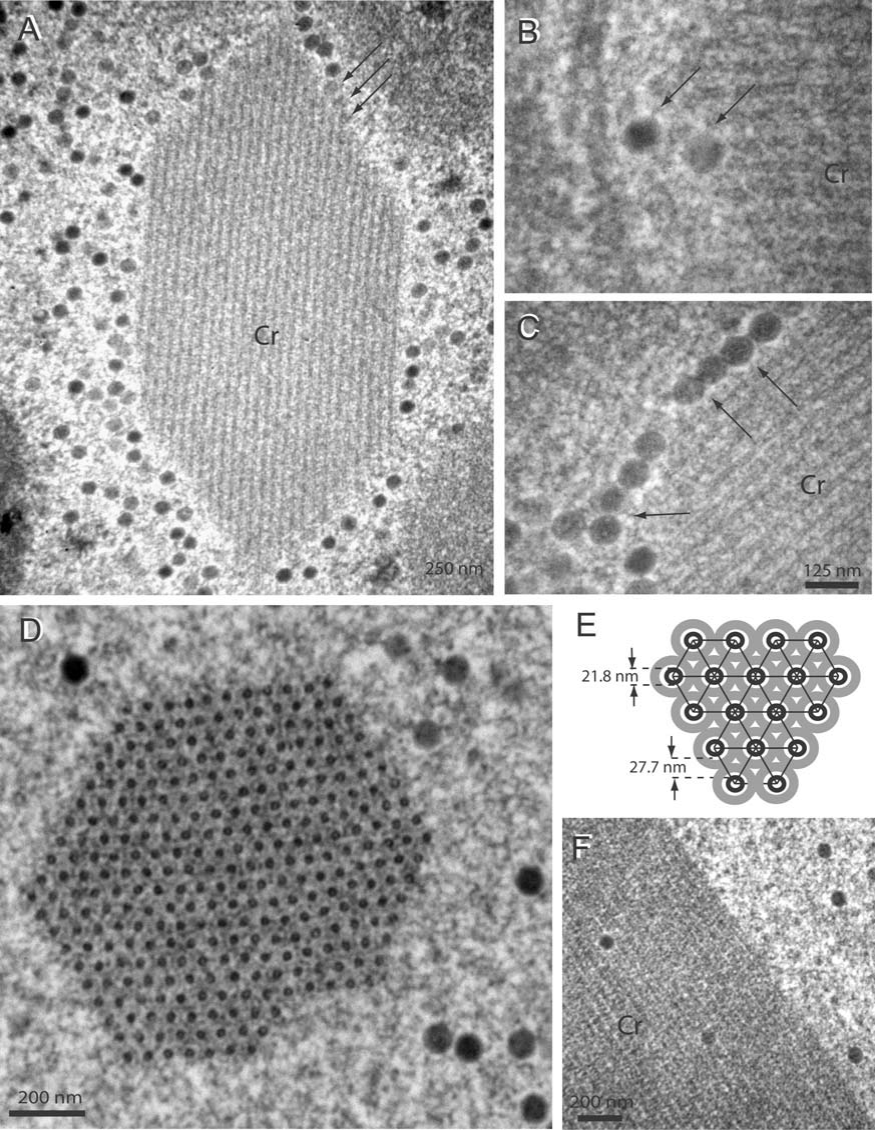
EM analysis of Ad5WTFib-infected 293 cells at 48 h pi. (A–C). Portions of cell nucleoplasm showing crystalline inclusions of viral proteins (Cr) seen in longitudinal sections. Note the presence of virions in close contact with crystal elements. Arrows on top of panel (A) indicate virions which seem to emerge from the edge of the crystal. Arrows on panel (B) and (C) point to filaments connecting virions to the crystal. (D), Portion of cell nucleoplasm showing a viral protein crystalline inclusion seen in cross section. (E), Model of the crystal lattice viewed in cross section, with its main parameters, diameter of the tubular unit (TD = 21.8) and inter-tubular distance (IT = 27.7), indicated in nm. In (F), a longitudinally sectioned crystal showed adenovirions included within the crystal lattice. Epon-embedded specimens.

The assembly pattern of Ad5 and nuclear alterations induced by the viral infection was examined *in situ* by EM, using HEK-293 cells mock-infected or infected with Ad5WTFib. At 48 h pi, the pattern of intranuclear viral material was the one expected from earlier studies on WT Ad-infected cells. It consisted of virions with their typical electron-dense core and of crystalline inclusions of viral proteins (Fig. 1 A). These crystals have previously been described and their structural parameters determined in Ad2-infected KB cells [21], [25]. In longitudinal and cross-sections observed under different angles (Fig. 1 A–D), they appeared to be formed by the regular arrangement of a pair of concentric tubules of two different shades, a dark inner tubule surrounded by a lighter shaded one (Fig. 1 E ; see also [25]). The diameter of the dark inner tubule (TD) was 21.81±2.69 nm (mean±standard deviation, SD; n, number of independent measurements in different areas of crystal sections = 29), and the inter-tubular distance (IT) was 27.70±2.74 (mean±SD; n = 131). In many cases, (Fig. 1 A, arrows), there was a contiguity, and even a continuity of electron-dense material between protein crystal elements and virions, in particular at the tip of some tubules. Contacts between virion and crystal material were also materialized by thin filaments connecting the Ad capsid to tubular elements of the crystal (Fig. 1 B, C; arrows).

### Immuno-EM analysis of Ad5WTFib-induced protein crystalline inclusions

**Figure 2:**
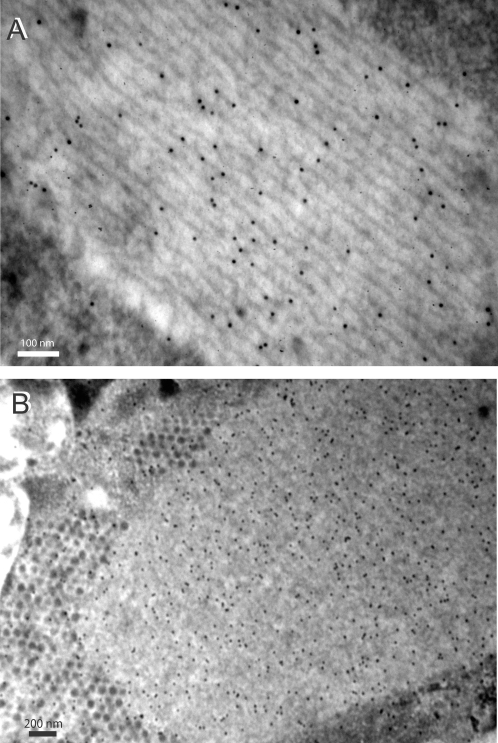
EM and immuno-EM analysis of Ad5WTFib-infected 293 cells at 48 h pi. Sections of metacrylate-embedded specimens were reacted with rabbit anti-fiber (A), or anti-penton base antibody (b), followed by 10-nm colloidal gold-labeled anti-rabbit IgG goat antibody. Shown are portions of nucleoplasm with protein crystalline inclusions. In panel (A), the longitudinal section of the protein crystal shows gold grains disposed along the lines of the tubular structures. In (B), crystalline inclusions of virus particles and viral protein are seen in close contact with each other, a phenomenon which is called epitaxy. Note that fixation and staining of metacrylate-embedded specimens was deliberately weak, in order not to interfere with the immunogold labeling.

The nature of the viral protein components of the intranuclear crystals was investigated using immuno-EM. Ad5WTFib-infected cell specimens were embedded in metacrylate resin, and sections reacted with various anti-Ad antibodies followed by 10-nm colloidal gold-tagged secondary antibody (immunogold labeling). A mixture of three monoclonal antibodies directed towards hexon group-specific epitopes in equal proportions, MAB8051, MAB8043 and 4C3, failed to react with intranuclear crystals in Ad5WTFib-infected cells (not shown). Likewise, no immunostaining was observed with anti-pIIIa, anti-core V and anti-core VII antibodies (not shown). With anti-fiber polyclonal antibody however, immunogold labeling concentrated in the crystalline inclusions, and in many instances the gold grains followed the linear arrangement of the tubules in longitudinal sections of crystals (Fig. 2 A). Antibody against penton base gave an immuno-EM pattern similar to that of anti-fiber, with a roughly linear arrangement of immunogold grains along the crystal tubules, as with anti-fiber antibody (Fig. 2 B). This indicated that the intranuclear protein inclusions observed at late times of Ad5 (or Ad2) infection were composed of penton capsomers, a hetero-oligomeric protein complex composed of penton base capsomer bound to the projecting fiber.

### Characterization of TB5, an anti-crystal monoclonal antibody

In order to confirm the protein composition of the nuclear crystals, Ad5WTFib-infected 293 cells were also studied by conventional and confocal immunofluorescence (IF) microscopy. A mouse monoclonal antibody, named TB5, was raised against Ad5-infected cell lysate. TB5 was found to be directed against native fiber, as it reacted with fiber in immunoprecipitation of (^35^S)-methionine-labeled Ad5-infected Hep2 cell extracts, but not with SDS-denatured fiber in Western blots, indicating that the TB5 epitope is in a sensitive three-dimensional conformation (data not shown). The reactivity to TB5 was compared between standard 293 cells and its derivative, the fiber-expressing cell line 293-Fiber [7]. IF analysis showed an intense and diffuse fluorescent signal in 293-Fiber cells, mainly localized in the nucleus, whereas only background fluorescence was observed with 293 cells (data not shown). This confirmed that TB5 was a *bona fide* anti-fiber antibody. Furthermore, nuclei of Sf9 cells infected by recombinant baculovirus AcMNPV expressing Ad5, Ad2 or Ad3 fiber knob [29], also reacted with TB5, whereas uninfected Sf9 did not yield a detectable signal (data not shown). This indicated that the TB5 epitope was localised in the knob domain, and was common to Ad2, Ad5 and Ad3 serotypes.

**Figure 3:**
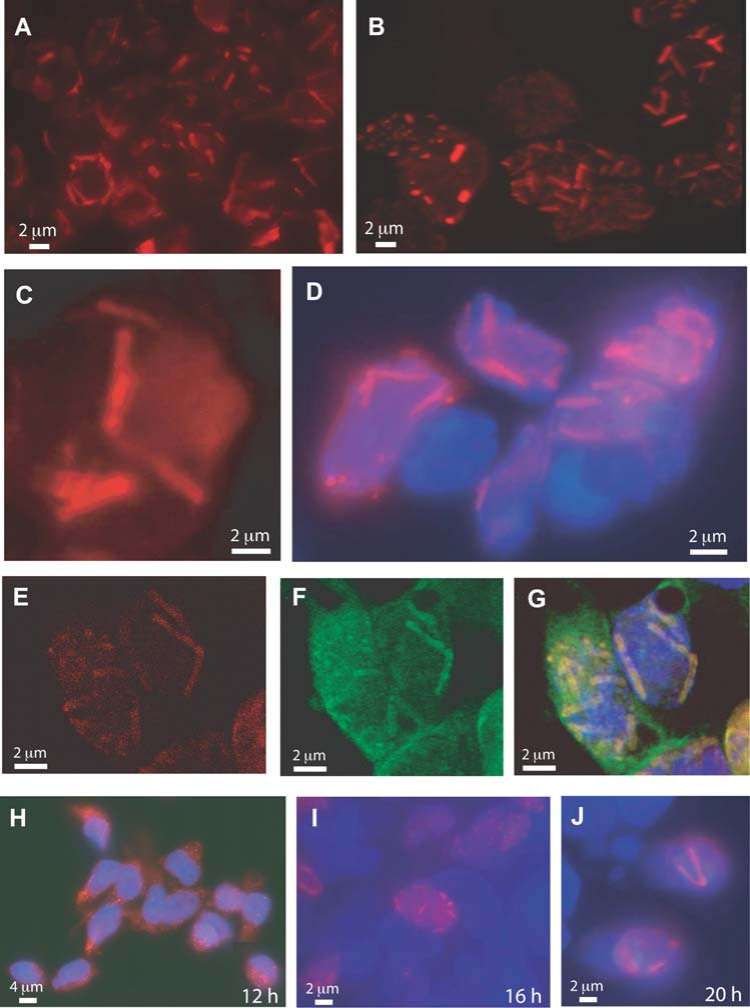
IF microscopy of Ad-infected 293 cells at 48 h pi. (A–D) and (H–J). Conventional IF microscopy using anti-fiber monoclonal antibody TB5 and secondary Alexa Fluor®633-labeled anti-mouse IgG. (A), Ad5WTFib; (B), Ad5PbEGD; (C), Ad5H508A; (D), Ad5(HI)RGD4C (with DAPI counterstaining). (E–G), Confocal analysis of Ad5ßGalWTFib-infected 293 cells with TB5. (E), Sample reacted with TB5 Alexa Fluor®633-labeled anti-mouse IgG; (F), sample reacted with anti-penton base antibody and secondary FITC-labeled anti-rabbit IgG; (G), image overlay with DAPI nuclear staining. (H–J), Kinetics of appearance of intranuclear crystals in Ad-infected cells. 293 cells infected with Ad5WTFib were harvested at different times pi and reacted with TB5 and Alexa Fluor®633-labeled anti-mouse IgG, with DAPI counterstaining. (H), 12 h pi; (I), 16 h pi; (J), 20 h pi. Note that IF pictures are presented at low magnification to show large field of view including several crystal-containing cells (A, B ; H–J), or at higher magnification to show the mass and number of crystals within a single nucleus (C–G).

**Figure 4:**
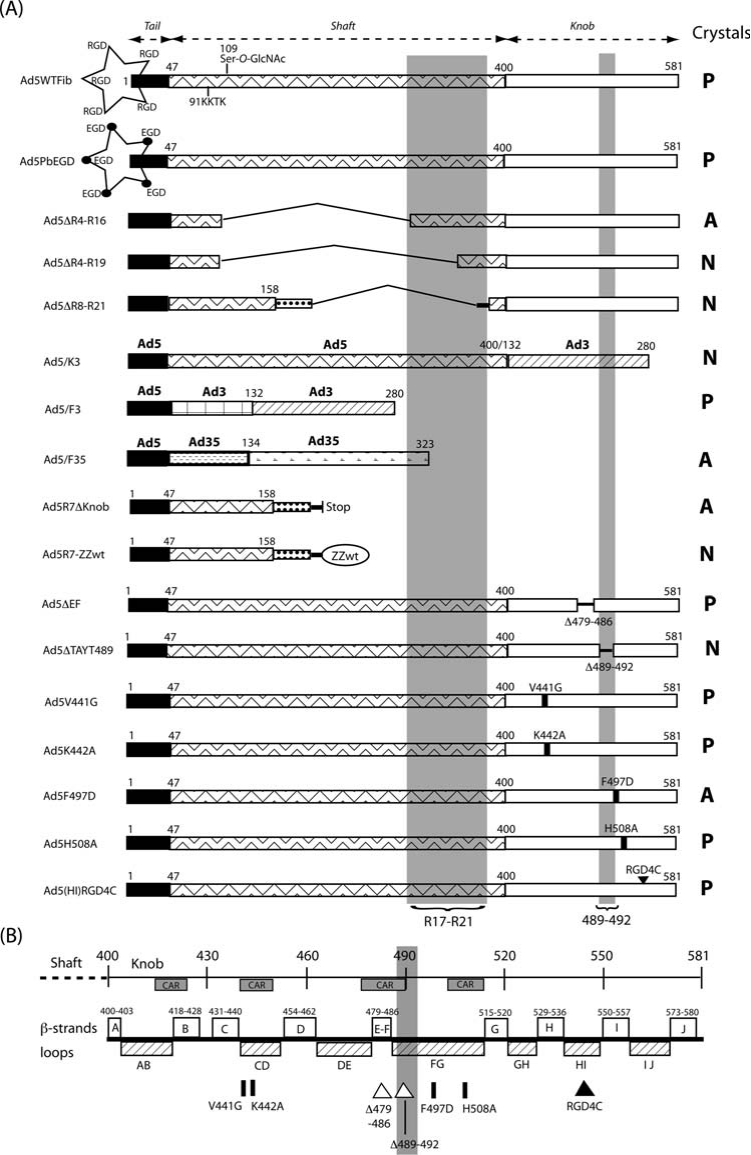
(A), Schematic representation of penton base and fiber mutant constructs in Ad5 vectors. The acronyms of the recombinants are indicated on the left side of the figure. The penton base and its RGD loops is represented by a five pointed star. The different structural domains and ligands of the fibers are shown by various symbols, as indicated in the figure. In Ad5ΔR8-21 and Ad5R7ΔKnob fibers, the extrinsic trimerization motif is represented by a stippled bar. On the right side of the figure, ‘Crystals’ indicates the occurrence of nuclear protein crystals : P, positive for crystals; N, negative; A, altered in crystal lattice arrangement. The shaded areas represent the regions of the fiber knob and shaft domains which are crucial for crystal formation. (B), Schematic representation of the Ad5 fiber knob domain and mutation positions. The end of the shaft domain is represented by a dotted line on the left side, and the knob domain by a solid line, with the amino acid numbering starting at residue 400. The β-strands regions are represented as open boxes, the flexible loops as hatched boxes, and the regions involved in CAR receptor binding [31] as shaded boxes. The positions of mutations are indicated by solid bars for substitutions, by open triangles for deletions, and by a solid triangle for the RGD4C insertion.

Ad5-infected 293 cells harvested at late times pi (48 h), fixed, permeabilised and incubated with TB5 showed intensely-labeled fluorescent inclusions in nuclei in conventional IF microscopy (Fig. 3 A–D), reminiscent of the intranuclear crystals detected by EM. Confocal microscopy confirmed the regular, rod-like structure of these inclusions (Fig. 3 E). More importantly, the intranuclear rod-like inclusions were simultaneously labeled with both TB5 and anti-penton base antibody (Fig. 3 E–G). DAPI stained the nucleoplasm in a diffuse manner and its blue fluorescent signal was excluded from crystals (Fig. 4 D, G). This indicated that DNA was not a constitutive element of the crystals, confirming previous histochemical analyses [30]. When compared to polyclonal anti-fiber antibody, the reactivity of monoclonal TB5 towards crystal sections was very weak in immuno-EM, as expected for a single epitope-recognizing monoclonal antibody, but some immunogold labeling was found to be associated with crystalline inclusions (not shown).

### Involvement of penton base and fiber domains in the crystal structure

HEK-293 cells were infected at the same MOI with a panel of recombinant Ad5 carrying penton base or fiber mutations (schematically represented in Fig. 4), processed for EM, and examined at 48 h pi. We reasoned that if the intranuclear protein crystals formed at the late stage of Ad5 infection were composed of pentons, certain mutations in one or the other domain of the protein, penton base, fiber shaft or fiber knob, might affect the occurrence and/or structure of the crystals and hopefully result in structural alterations visible under the EM. The level of viral infection was assessed under the EM by the occurrence of numerous intranuclear virions isolated or packed in clusters (refer to Fig. 1 and 2).

**Figure 5:**
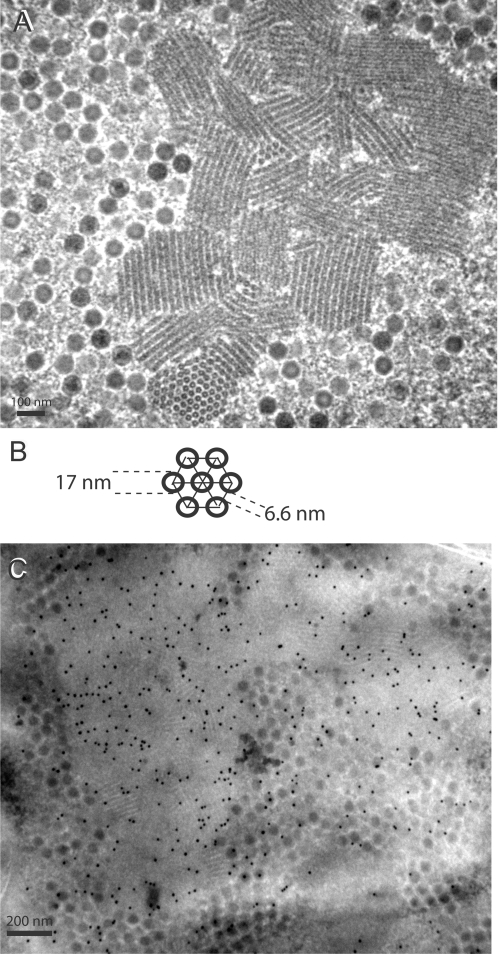
EM (A), and immuno-EM (B) analyses of Ad5R7Δknob-infected 293 cells at 48 h pi. In panel (A), an area of nucleoplasm shows small and twinned protein crystals seen in longitudinal, oblique and cross-sections. Virions are seen dispersed in the neighbourhood. Epon-embedded specimens. In panel (B), is shown a model of the crystal lattice derived from data obtained from as in (A). In panel (C), a section of metacrylate-embedded specimen was reacted with rabbit anti-penton base antibody followed by 10-nm colloidal gold-labeled anti-rabbit IgG goat antibody. Note the accumulation of gold-labeling in the protein crystalline inclusions. Some gold grains are also seen associated with virion clusters.

Mutant Ad5PbEGD, which carried a R-to-E substitution in the penton base RGD motif, induced the formation of nuclear protein crystals with WT characteristics as judged by EM (not shown) and showed the same pattern of nuclear inclusions following incubation with TB5 (Fig. 3 B), indicating that the formation of the crystalline inclusions did not depend on the integrity of the RGD motifs of penton base capsomers. However, Ad5R7Δknob, a knob-deleted mutant carrying fibers with seven shaft repeats and no cell-specific ligand (Fig. 4), showed a drastic change in the crystal morphology, compared to WT crystals : crystals generated by Ad5R7Δknob were more compact, arranged as twinned crystals or as macles (Fig. 5 A). The dark tubules constituting the crystals were shorter than in WT crystals, and the grey area around them (refer to Fig. 1 D, E) was no longer visible. There was a lower value for the inner tubule diameter (TD = 17.3±1.94 nm ; n = 15) and a significantly smaller (3-fold) distance between tubules (IT = 6.60±1.59 nm; n = 28) (Fig. 5 B). When examined in immuno-EM using penton base antibody, Ad5R7Δknob crystals showed a positive response, but a less regular arrangement of gold grains than in WT crystals (Fig. 5 C). This suggested that penton base protein constituted the inner tubules of the crystals, whereas the peripheral grey area would represent the fiber moiety of the penton heteromeric protein. As expected, the Ad5R7Δknob-infected cell nuclei did not show any reaction with TB5 in IF (data not shown).

### Mapping of the fiber knob region(s) implicated in the crystal structure

**Figure 6:**
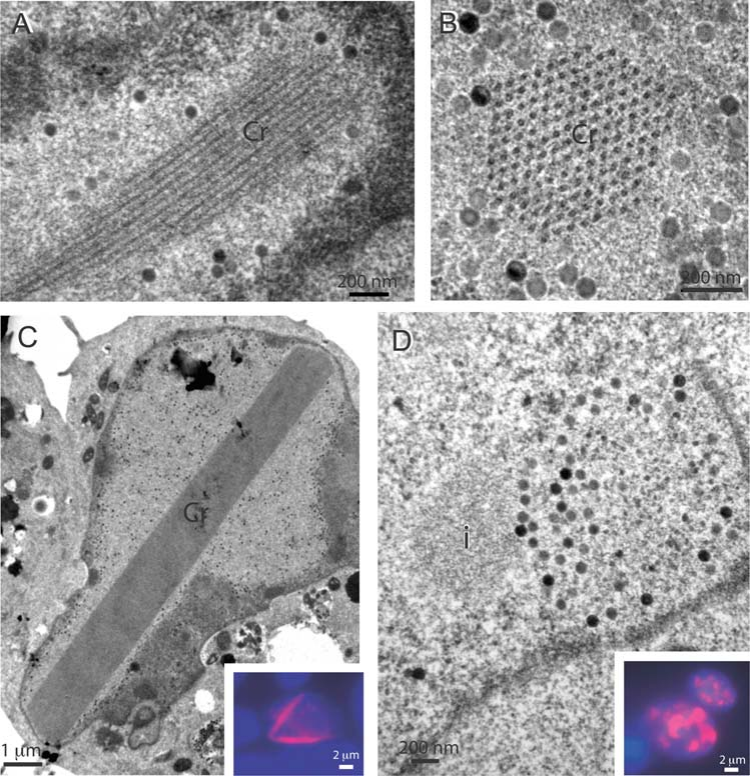
EM analysis of 293 cells infected with Ad fiber knob mutants at 48 h pi. (A–C), Ad5ΔEF crystals (Cr) seen in longitudinal (A, C) and cross section (B); (C, inset), Ad5ΔEF-infected cells examined in IF microscopy after reaction with TB5 followed by Alexa Fluor®633-labeled anti-mouse IgG and DAPI counterstaining. (D), Ad5ΔTAYT489; (i), amorphous inclusion. (D, inset), Ad5ΔTAYT489-infected cells stained with TB5, as for the specimen shown in the inset of panel C.

In order to define the region(s) of the knob domain which were critical for crystal assembly, we used Ad5 mutants carrying substitutions, short deletions or insertions in their knob domain (refer to Fig. 4 B). Most of these mutations concerned accessible residues involved, directly or indirectly, in CAR binding of Ad5 or Ad12 fiber, e.g. at positions 441, 442 and 508 (reviewed in [31]). Substitution mutants Ad5V441G, Ad5K442A and Ad5H508A showed a WT pattern of intranuclear crystalline inclusions (Fig. 3 C), as well as insertion mutant Ad5(HI)RGD4C (Fig. 3 D). Deletion mutant Ad5ΔEF, which lacked residues 479–486 forming the short double beta-strand EF [32], [33], also showed TB5-stained intranuclear crystals indistinguishable from that of WT in fluorescence microscopy (Fig. 6 C, inset). Under the EM, Ad5ΔEF-induced nuclear crystals presented overall WT features (Fig. 6 A–C). However, closer examination at higher magnification revealed subtle differences in certain crystal parameters compared to those of WT crystals. The inner tubule diameter was similar in size to that of WT (TD = 23.20±3.69 nm; n = 20), but the intertubular distance was slightly shorter, IT = 23.51±3.29 (n = 20), instead of 27.7±2.74 nm for WT crystals (refer to Fig. 1 A–E). In contrast, the nuclei of Ad5ΔTAYT489-infected cells showed no detectable crystals and only amorphous inclusions were observed (Fig. 6 D). In IF microscopy of Ad5ΔTAYT489-infected cells, TB5-stained globular inclusions were observed (Fig. 6 D, inset).

**Figure 7:**
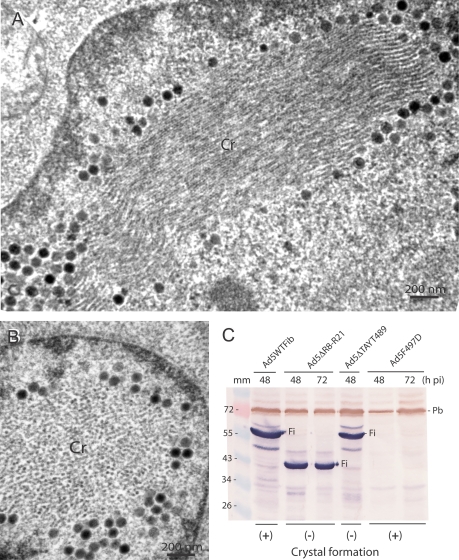
Occurrence of intranuclear crystals and fiber protein expression in cells infected with Ad5 fiber mutants. (A, B), EM analysis of Ad5F497D-infected cells at 48 h pi. Cr, crystal. (C), SDS-PAGE and Western blot analysis of whole lysates from cells infected respectively with : Ad5WTFib, Ad5ΔR8-R21, Ad5ΔTAYT489 and Ad5F497D, harvested at 48 or 72 h pi as indicated on top of the panel. Blots were reacted with anti-tail mAb 4D2.5 followed by phosphatase-labeled anti-mouse IgG antibody, then with anti-penton base rabbit antibody and peroxidase-labeled anti-rabbit IgG antibody. Fi, fiber polypeptide band; Pb, penton base. Symbols (+) and (−) at the bottom of the blot refer to Ad5 clones which were positive or negative for crystal formation, respectively.

Mutant Ad5F497D was mutated in the first residue of the highly conserved motif FMP [34]. The F-to-D substitution at position 497 has been shown to have deleterious effects on fiber encapsidation, virus maturation and infectivity of Ad5F497D mutant [19]. We therefore examined Ad5F497D-infected 293 cells under the EM, and found that the structure of the intranuclear inclusions induced by Ad5F497D was drastically different from that of WT, both qualitatively and quantitatively. Qualitatively, the tubules forming the crystals had a wavy aspect in longitudinal sections (Fig. 7 A), and showed irregularly arranged rings in cross sections (Fig. 7 B), compared to WT crystals (refer to Fig 1 D). Quantitatively, the crystal parameters had also changed, compared to those of WT : tubule diameter and intertubular distance were both significantly smaller than to those of WT crystals, TD = 16.86±2.72 (n = 8), and IT = 21.87±2.45 (n = 23), respectively. In IF microscopy with TB5, no fluorescent signal was obtained with Ad5F497D mutant (not shown).

Although these data suggested *a priori* that the knob region overlapping the 489-TAYT-492 and F-497 mutations was essential for crystal formation and/or correct lattice structure, we envisaged the possibility that the absence of crystals, or their aberrant structure, could be related to the amount of fiber protein in mutant-infected cells. Whole lysates from cells infected with Ad5F497D and Ad5ΔTAYT489 were therefore analyzed by SDS-PAGE and Western blotting using anti-tail 4D2.5 mAb, and their fiber content compared to that of Ad5WTFib and of Ad5ΔR8-R21, another mutant defective in crystal formation (see below). Penton base protein was also assayed by Western blot analysis and used as the internal standard. Fiber production was found to be at WT levels for Ad5ΔR8-R21 and Ad5ΔTAYT489, but barely detectable for Ad5F497D, even at late times pi (Fig. 7 C), suggesting a lower level of expression or/and a lower stability of the F497D fiber protein mutant, compared to WT fiber. The low cellular content of F497D fiber correlated with the low fiber copy number of Ad5F497D virus progeny and the low infectivity of virions previously observed [19]. By contrast, the penton base production was at WT levels at 72 h pi in Ad5F497D-infected cells (Fig. 7 C), implying that the aberrant inclusions generated by Ad5F497D mutant contained no fiber protein and only penton base. However, the normal fiber content of cells infected with Ad5ΔTAYT489 and Ad5ΔR8-R21 mutants indicated that the absence of crystals was not the direct consequence of a low level of fiber protein expression.

Except for the fiber mutant F497D described above, and the two knobless fibers R7Δknob and R7-ZZ_wt_ which were defective in fiber protein synthesis [17], [18], all the other fiber mutants used in the present study were produced at levels similar to that of WT fiber (data not shown).

### Role of the fiber shaft domain in crystal formation and structure

**Figure 8:**
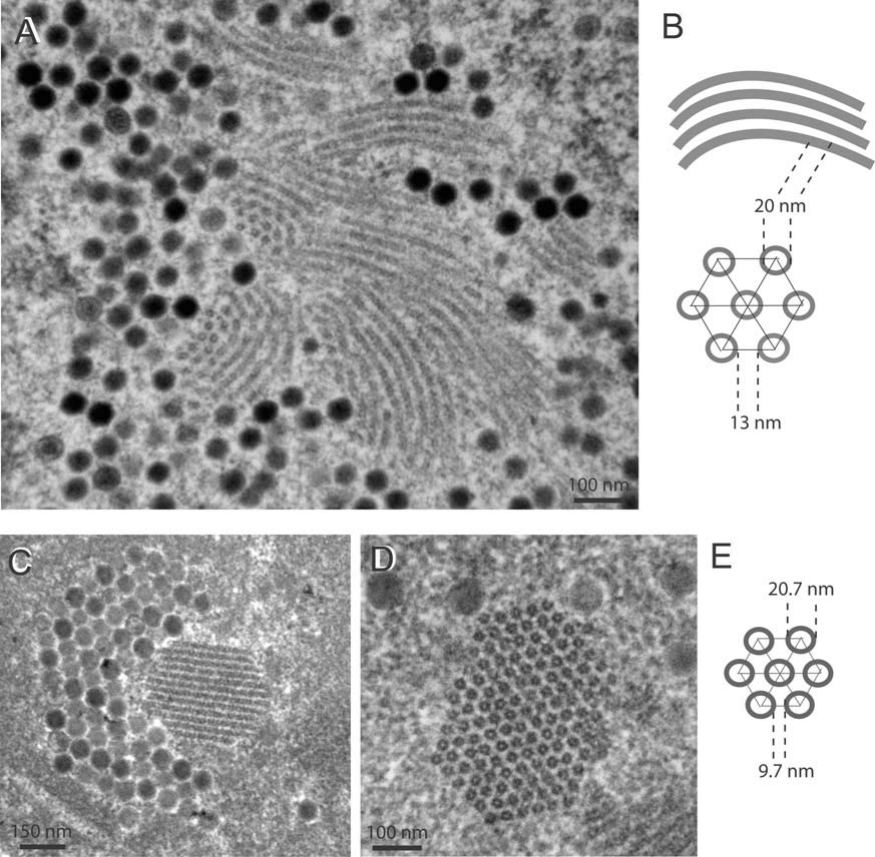
EM analysis of 293 cells infected with (A, B) shaft-deleted recombinant Ad5ΔR4-R16, or (c–e) chimeric fiber recombinant Ad5/F3, at 48 h pi. In panels (A) and (C, D), areas of nucleoplasm show small, twinned protein crystals seen in both longitudinal and cross-sections. Panels (B) and (E) present models of the two types of crystal lattice.

We next examined whether the size and nature of the fiber shaft had any influence on the structure of the crystals. Three deletions were made in the fiber shaft, spanning repeats 4 to 16, 4 to 19 and 8 to 21, corresponding to viruses Ad5ΔR4-16, Ad5ΔR4-19 and Ad5ΔR8-21, respectively (Fig. 4). Of note, R4-R16 and R4-R19 deletions included the *O*-GlcNAc-serine residue at position 109 [35] in the fourth shaft repeat, but respected the 91-KKTK-94 motif in the third repeat, which contains a bend in the shaft rod-like structure [36]. Ad5ΔR4-16 produced crystals, although with different shape and dimensions, compared to WT crystals (Fig. 8 A, B) : in longitudinal sections the tubules showed a certain degree of curvature; in transverse sections, the tubules were similar to WT in diameter, but inter-tubular spacing was narrower than in WT crystals. Measurements gave the following values: TD = 20.17±2.05 (n = 18), and IT = 13.18±3.82 (n = 20). No crystal was observed with Ad5ΔR4-19 and Ad5ΔR8-21, and only amorphous inclusions could be seen under the EM (not shown). This suggested that the shaft region overlapping repeats 17–21 was critical for intranuclear crystallization process.

The role of the shaft domain was further analyzed using chimeric penton and fiber capsomers. Ad5/K3 carried only the Ad3 knob at the extremity of a serotype 5 fiber bound to serotype 5 penton base, whereas Ad5/F3 carried the Ad3 knob and shaft domains fused to the tail of Ad5 fiber. In Ad5/F35, the virion was pseudotyped by serotype 35 fiber knob and shaft, fused to serotype 5 tail (Fig. 4). No crystal was observed in Ad5/K3-infected 293 cells (not shown). However, Ad5/F3 produced crystals with a particular morphology (Fig. 8 C–E) : crystalline inclusions were generally much shorter than with Ad5WTFib or Ad5ΔEF (compare with Fig. 1 A, F and Fig. 6 C). Tubular diameter was in a similar range of values as in WT crystals (TD = 20.74±1.80 ; n = 15), but the intertubular distance was significantly smaller, ca. 3-fold (IT = 9.71±0.50 ; n = 48).

**Figure 9:**
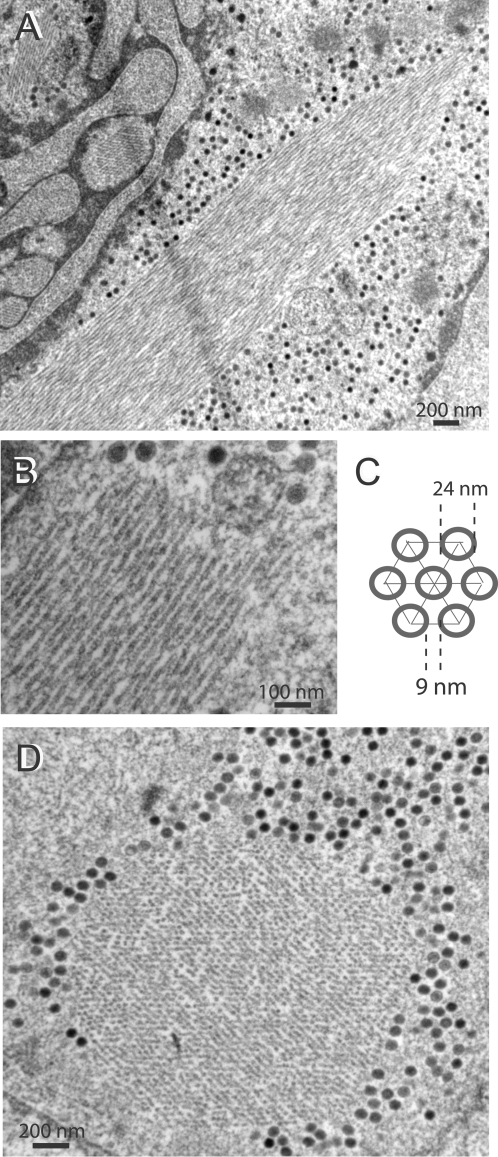
EM analysis of 293 cells infected with chimeric fiber recombinant Ad5/F35, at 48 h pi. Protein crystals are shown in (A, B) longitudinal sections at low (A) and high (B) magnification, and (D) cross-section. (C), Model and parameters of the crystal lattice.

The morphology of crystals produced by the pseudotyped vector Ad5/F35 was also different from the WT crystals (Fig. 9) : the tubular alignment was not perfectly straight as in WT crystals (Fig. 9 A), and the tubules showed very frequent discontinuities in longitudinal sections (Fig. 9 B). The tubule diameter was slightly larger and the spacing narrower than those of WT: TD = 24.32±2.60 (n = 91) and IT = 9.26±3.48 (n = 73).

In IF microscopy with TB5, no fluorescent signal was obtained with the pseudotyped vector Ad5/F35, and a diffuse pattern of fluorescence was shown by the shaft deletion mutants Ad5ΔR4-19 and Ad5ΔR8-21, as well as with the pseudotyped vector Ad5/K3 (not shown).

### Occurrence of protein crystals in cells infected with dual fiber-expressing vector Ad5/R7-ZZ_wt_/E1:WTFib

In a previous study, we have shown that the deletion of the knob had a negative effect on the rate of translation of the fiber mRNA, resulting in a low cellular content of knobless fiber, compared to WT fiber. In addition, knobless fiber Ad5 vectors have a lower fiber encapsidation efficiency and lower infectivity than WT fiber vectors [18]. To further examine the cellular effects of the coexpression of two fiber proteins, WT and knobless, in the virus assembly process, we infected 293 cells with a single fiber-expressing or a dual fiber-expressing vector, and examined cell sections under the EM at 48 h pi. Ad5/R7-ZZ_wt_ contained a single fiber gene at its natural location in the L5 region of the adenoviral genome. Its fiber gene encoded a shaft-truncated (seven repeats; R7), knob-deleted fiber terminated by a non-viral trimerisation motif (abbreviated NRP) and a tandem Z_wt_ ligand [18], abbreviated ZZ_wt_ in the present study (Fig. 4 A). Fiber trimerization of knobless R7-ZZ_wt_ fiber was therefore achieved by NRP, the neck region peptide from the human lung surfactant protein D [18]. The Z_wt_ ligand consisted of an Ig-binding domain derived from the *Staphylococcal* protein A, and has been described in previous studies [18], [37], [38]. In the dual fiber-expressing vector Ad5/R7-ZZ_wt_/E1:WTFib, the early region E1 located at the left-end hand of the viral genome was deleted and replaced by the gene coding for WT fiber, whereas the late region L5 encoded the knob-deleted, shaft-truncated ZZ_wt_-liganded fiber [18].

**Figure 10:**
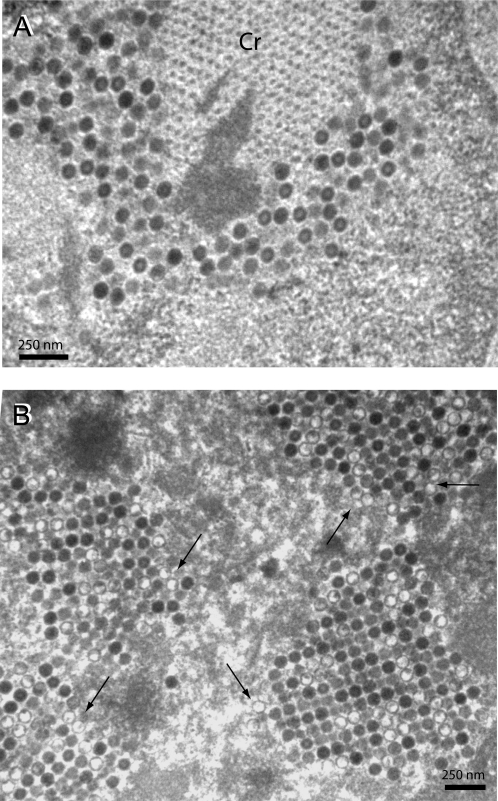
EM analysis of 293 cells infected with dual fiber-expressing recombinant Ad5/R7-ZZ_wt_/E1:WTFib. (A), Portion of nucleoplasm showing adenovirions clustering around a protein crystal (Cr) viewed in cross-section. (B), Another area of nucleoplasm showing Ad particles packed into clusters. Note that several particles have an electron-luscent centre (arrows).

Ad5/R7-ZZ_wt_-infected cells showed no protein crystalline inclusion, and many intranuclear virus particles showed an electroluscent center, representing empty capsids devoid of a DNA-containing core (not shown). This confirmed that the fiber knob domain, deleted in Ad5/R7-ZZ_wt_, was not only critical for efficient capsid assembly, but also for the formation of nuclear protein crystals, and possibly for DNA packaging into virions. In 293 cells infected with Ad5/R7-ZZ_wt_/E1:WTFib, two different patterns of virus particle assembly were observed : (i) clusters of virions with their usual shape and chromicity in close vicinity to protein crystals (Fig. 10 A); (ii) crystal-like packings of virus particles within the nucleoplasm at a distance from protein crystals, many of them showing an electroluscent center, typical of particles devoid of DNA (Fig. 10 B; arrows). The results of EM analysis of Ad5/R7-ZZ_wt_/E1:WTFib-infected cells suggested the coexistence of different modes of Ad assembly in double fiber-expressing cells, one of them involving the penton protein crystals.

**Figure 11:**
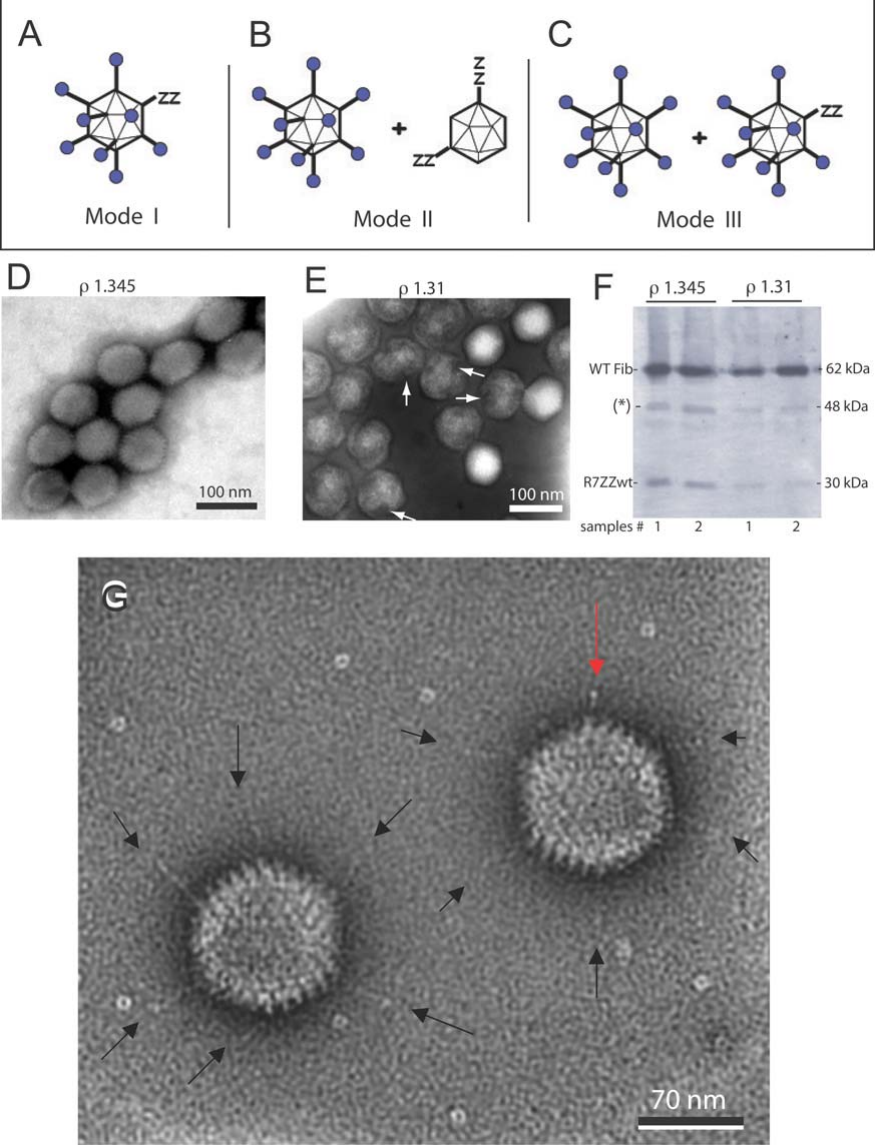
Analysis of the Ad5/R7-ZZ_wt_/E1:WTFib progeny. (A–C), Hypothetical modes of intranuclear assembly for Ad5/R7-ZZ_wt_/E1:WTFib virions. (A), Mode I : single population of mosaic fiber virions. (B), Mode II : two separate populations of virions, each carrying homogenous fiber species, WT and R7-ZZ_wt_ fiber, respectively. (C), Mode III : two separate populations of WT fiber virions and mosaic fiber virions, respectively. (D, E), EM analysis of Ad5/R7-ZZ_wt_/E1:WTFib virus progeny. (D), Virus population banding at a density (ρ) of 1.345 in CsCl isopycnic gradient. (E), Virus population banding at ρ = 1.30–1.31. White arrows point to gaps in viral capsids. (F), Fiber protein content of the virus population banding at the densities of 1.345 and 1.31, respectively. Virions were analyzed by SDS-PAGE and Western blotting using anti-tail mAb 4D2.5 followed by (^35^S)-labeled anti-mouse IgG antibody. An autoradiogram is shown. The band labeled with (*) at 48 kDa corresponds to the reaction of secondary anti-mouse IgG antibody with core protein V. (G), High resolution EM of Ad5/R7-ZZ_wt_/E1:WTFib progeny virions banding at ρ = 1.345. Black arrows point to long shafted, WT fibers. Red arrow points to a short shafted fiber.

Since two fiber protein species, WT and R7-ZZ_wt_, coexisted within the nucleoplasm, one could theoretically envisage three modes of assembly for Ad5/R7-ZZ_wt_/E1:WTFib virions (Fig. 11 A–C). (i) In the first mode (Fig. 11 A), there would be no preselection of one particular fiber species, and multiple equivalent assembly sites dispersed within the nucleoplasm would share the stock of available WT and R7-ZZ_wt_ fiber molecules. In this case, protein crystals would have no specific function in virus assembly, and the resulting virus progeny would consist of a single population of mosaic virions carrying the two fiber species in the same ratio as the cell content (Fig. 11 A). On the opposite, penton crystalline inclusions might play a role in the Ad morphogenic process and would represent a privileged assembly platform : in preselecting the WT fiber species, the crystals would provide the virus assembly machinery with a large supply of preassembled penton capsomers, the limiting factor in capsid assembly [39]–[41]. In this case, two possibilities could be considered. (ii) In the assembly mode II, WT fibers bound to penton base would exclusively localise in crystals, whereas the knobless fibers would be entirely left in other assembly sites invisible to conventional EM. This would yield a virus progeny composed of two different populations, each one homogenous in terms of fiber composition, WT or knobless R7-ZZ_wt_ fibers, respectively (Fig. 11 B). (iii) In the third assembly mode, WT fibers would not exclusively locate in crystals as penton base-bound molecules, but also be present in other assembly sites which would contain a mixed population of WT and R7-ZZ_wt_ fiber molecules. In this case, Ad5/R7-ZZ_wt_/E1:WTFib progeny would contain two types of virus particles, one carrying only WT fibers, the other consisting of mosaic particles carrying the two different fiber species (Fig. 11 C). Analysis of the Ad5/R7-ZZ_wt_/E1:WTFib progeny helped us to gain an understanding of the mechanism of Ad assembly within the nucleus.

### Characterization of the Ad5/R7-ZZ_wt_/E1:WTFib progeny

When analyzed by isopycnic ultracentrifugation in a CsCl self-generating gradient, the Ad5/R7-ZZ_wt_/E1:WTFib progeny was found to consist of two unequal populations : a major population (60–65% of the total virus yield, as determined by a conventional protein assay) sedimented as a broad band at an apparent density of ρ = 1.30–1.31, wheras the minor population (35–40%) banded at ρ = 1.340–1.345, the density of mature, infectious virions. Under the EM, negatively stained samples from the 1.345-band were found to consist of a homogenous population of virions with a typical icosahedral morphology (Fig. 11 D). By contrast, the band at 1.30–1.31 was heterogeneous and contained rare virions with a regular, icosahedral shape, and a majority of floppy particles lacking a polyhedral contour (Fig. 11 E). Gaps in the capsid were clearly observed at the expected positions of apices (Fig. 11 E; arrows). This aspect was in line with the EM observations of Ad5/R7-ZZ_wt_/E1:WTFib-infected cell nuclei (refer to Fig. 10) and implied that a large population of the virus progeny consisted of incomplete, non-infectious particles devoid of viral genomes. This was confirmed by the determination of the infectious titre and DNA content, which were were about 10-fold lower in the 1.30-particle fraction, compared to the 1.34-fraction. Interestingly, both fractions were found to contain the two fiber species, with a WT to R7-ZZ_wt_ ratio of about 10∶1, as shown by Western blot analysis (Fig. 11 F).

We next examined at high resolution EM the Ad5/R7-ZZ_wt_/E1:WTFib particles banding at 1.34-density, since these particles showed intact apices, by contrast to 1.30-particles. EM analysis was consistant with the Western blot data and ruled out modes I and II. Both fiber species, WT and knobless, were found in particles of 1.34-density, and we never observed virions carrying solely short, knobless fibers (Fig. 11 G). Although the twelve fiber projections are rarely seen simultaneously due to their masking by the capsid, two populations of intact virions were clearly distinguished. The major population consisted of virions with their apical capsomers occupied by WT fibers, with their typical shape, size, and terminal knob (Fig. 11 G; black arrows). The minor virus population showed a mixed fiber content, with coexistence of long, knob-carrying fibers (Fig. 11 G ; black arrows) and short, knobless fibers on the same capsid (Fig. 11 G; red arrow). This was in favor of the assembly mode III (Fig. 11 C).

**Figure 12:**
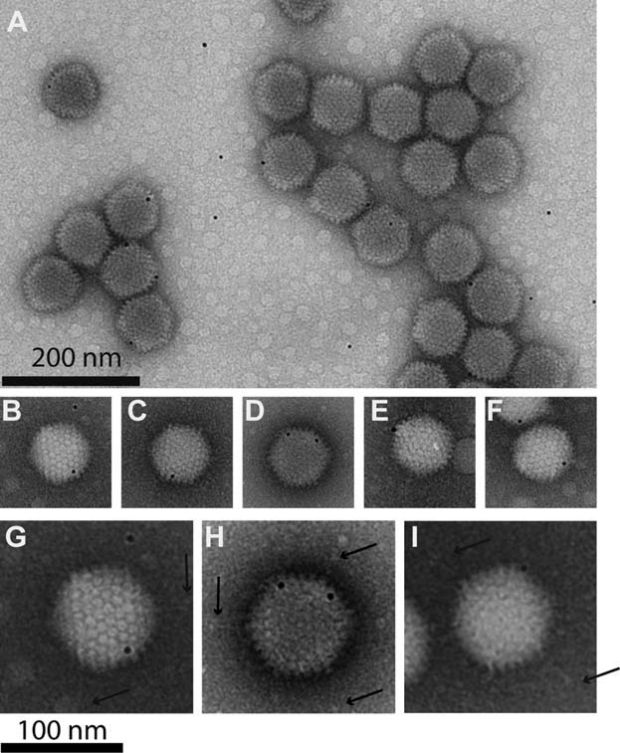
EM analysis of Ad5/R7-ZZ_wt_/E1:WTFib virus particles after immunogold labeling. Ad5/R7-ZZ_wt_/E1:WTFib particles of 1.34 in density were incubated with 6-nm gold-tagged polyclonal IgG from goat, and examined under the EM after negative staining with ammonium molybdate. The short, knob-deleted fibers R7-ZZ_wt_ were visualized by 6-nm gold-tagged goat IgG binding to the tandem ZZ_wt_ motif (IgG-Fc ligand). Long shafted WT fibers with their terminal knob are indicated by arrows.

WT and R7-ZZ_wt_ fibers could be distinguished not only by their length and knob domain, but also by their immune reactivity. Since the ZZ_wt_ tandem motif is a ligand of the Fc domain of IgG [37], [42], the Ad5/R7-ZZ_wt_/E1:WTFib particles of 1.34 in density were incubated with gold-tagged polyclonal IgG from goat, and examined under the EM. Immunogold labeling confirmed the heterogeneity of this virus population in terms of fiber content, as described above. The majority of virus particles were unlabeled and carried only WT fibers. They coexisted with another population of virus particles which were immunogold labeled and represented 25–30% of the total (Fig. 12 A). Most of the virus-associated gold grains were found to localize at the apex of the capsid (Fig. 12 B–F). Observation of isolated virus particles at higher magnification showed the coexistence of two fiber species on single capsids, long WT fibers terminated by the knob (Fig. 12, G–I; arrows), and gold-tagged R7-ZZ_wt_ fibers (Fig. 12, G–I). This further supported the assembly mode III proposed in Fig. 11 C.

### Time-course kinetics of intranuclear occurrence of penton crystals in Ad5WTFib-infected cells

If intranuclear penton crystals represented byproducts from viral capsid protein synthesis, one would expect that the appearance of crystals would be delayed in Ad-infected cell nucleus, compared to that of virus particles, and would constitute a very late event in the virus life cycle. On the opposite, if crystals were directly or indirectly involved in virion assembly, the timing of their occurrence should roughly coincide with the step of adenovirion assembly [41]. Taking advantage of the TB5 reactivity of crystals *in situ*, 293 cells were infected with Ad5WTFib and samples withdrawn at regular time intervals for IF microscopy with TB5 staining. A diffuse cytoplasmic fluorescence was observed at 12 h pi, consistent with the start of the fiber biosynthesis at this phase of the cycle (Fig. 3 H). Intranuclear fluorescent dots and rod-like inclusions were seen as early as 16 h pi in 2–5% of the infected cells (Fig. 3 I), and typical crystals were visible at 20 h pi in 10% of the cells (Fig. 3 J). At later times pi, more than 50% cells showed large intranuclear crystals (refer to Fig. 3 A). This indicated that the occurrence of intranuclear cystals was contemporary with the start of virion assembly (16–20 h pi), and would exclude the crystals as surplus material from capsid building. It rather suggested that crystals played a role in virus particle morphogenesis.

## Discussion

### Nature of the protein crystalline inclusions observed in Ad-infected cells

The protein composition of the crystalline inclusions induced by Ad5 (or Ad2) in the nucleus of infected cells at late times after infection has been long debated [21], [23]–[27], and their role in the Ad life cycle has not been elucidated. Their structural characteristics and the contiguity between crystals of virions and crystals of proteins [21], [22], [26], a phenomenon termed epitaxy [25], led to the hypothesis that these crystals were constituted of the major capsid proteins, hexon, penton base and fiber [21], [25]. In line with this hypothesis, previous investigators provided structural description of the crystals similar to that presented in this study, and morever, by using temperature-sensitive (*ts*) mutants, suggested that the crystals were derived from fibers, with a proviso that penton base might also be involved [28]. However, other studies suggested that the crystals were formed of basic core proteins [23], [24], [26]. More recent immuno-EM analyses revealed that the intranuclear crystals reacted with anti-hexon, anti-penton base and anti-fiber knob antibodies [43], as well as with protein kinase CK2 and dsRNA-activated protein kinase PKR antibodies, leading to the assumption that multiple viral and cellular proteins were components of the crystals [44].

The reactivity of nuclear crystals with hexon antibodies was in contradiction with two previous genetic studies. (i) In cells infected with Ad5*ts*27, a mutant defective in hexon expression at the nonpermissive temperature 38.5°C, intranuclear crystals were seen in a significant number of cells [28]. (ii) Interserotypic recombination between a crystal-producing strain and a non-producing strain of Ad2 has mapped the crystal-determining factor within 30 and 44 map units (m.u.) on the 100-unit Ad genome [45]. This region overlaps the L1 and L2 blocks of genes, and includes structural proteins IIIa (34.24 to 39.13 m.u.) and III (i.e. penton base; 39.37 to 44.14 m.u.), but excludes the genes coding for core proteins VII and V (44.16 to 45.82 and 46.02 to 49.10 m.u. in L2, respectively) and for hexon (52.41 to 60.50 m.u. in L3) [46].

The reactivity of nuclear crystals with cellular protein antibodies was also in contradiction with some basic principles of protein biochemistry. Protein crystallisation is considered to be the best criterion for protein purity and homogeneity, and there is no reason for intracellular crystals being an exception to this rule. It could not be excluded however, that some viral or host cell proteins might be trapped in growing crystals, without being *bona fide* crystal components. This was the case for Ad virions, which were sometimes found trapped within the crystal lattice (Fig. 1 F), a phenomenon reminiscent of baculovirus particle inclusion within polyhedrin crystals [47], [48].

In the present study, we showed that protein crystals induced by WT Ad5 in HEK-293 cells did not react in IF or immuno-EM with anti-hexon, anti-pIIIa, anti-core V, or anti-core VII, but only with penton base and fiber antibodies. The crystals were highly reactive in IF with TB5, a monoclonal antibody generated against Ad5 fiber, and whose epitope was located in the knob domain. We could conclude that the intranuclear crystalline inclusions occurring in Ad-infected cells were formed of penton capsomers, a hetero-oligomeric protein complex composed of two moieties, a penton base homopentamer and a fiber homotrimer.

A definite demonstration of the role of penton base and fiber as the only viral proteins responsible for crystal formation would be the occurrence of crystals in baculovirus-infected insect cells coexpressing these two protein partners. However, only amorphous inclusions were observed in the nucleus of Sf9 cells coinfected by baculoviruses expressing Ad2 penton base and fiber, although penton indistinguishable from penton capsomer isolated from human cells was recovered from Sf9 cell lysates [49]. This would suggest that penton crystal formation might be influenced by the cellular context and require cellular protein(s) present only in mammalian cells. Alternatively and not exclusively, viral proteins other than penton base or fiber could play a role in the crystallogenetic process of penton crystals, e.g. in creating a nucleation center to initiate crystallization, or in the proper folding and conformation of intracellular penton molecules competent for crystallization.

### Protein domains required for crystallogenesis in Ad-infected cells

**Figure 13:**
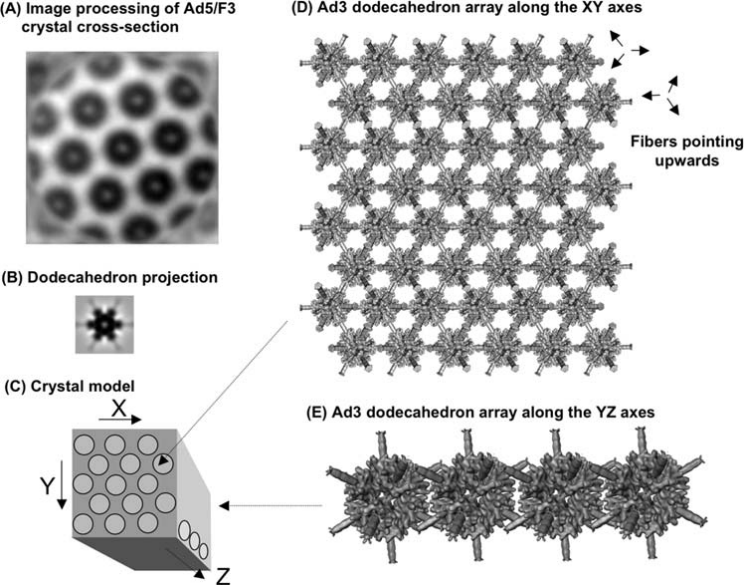
Model of the crystal lattice of Ad penton intranuclear inclusions. (A), 2D averaging of the Ad5/F3 crystal cross-section was generated from the crystal cross-section shown in Fig. 8 d. A total of 40 overlapping fields were cut out using the X3d program [72], and averaged after cross-correlation. (B), 2D projection of an Ad3 penton dodecamer (dodecahedron) with its 12 fiber projections, shown at the same magnification as in (a). The 3D map of a dodecahedron with 12 fibers was filtered to 45 Å and reprojected along its 3-fold axis using SPIDER [73]. (C), Schematic 3D representation of a portion of penton crystal, showing its three axes (arrows). (D), Dodecahedron array, presented along the XY axes. (E), Arrangement of the dodecahedron units along the YZ axes. The 3D isosurface representation shown in (D) and (E) was visualized using WEB [73].

In order to define which penton base or/and fiber subdomains were involved in the formation of intranuclear protein crystals, we examined their occurrence and structural features in cells infected by a panel of recombinant Ad5 carrying penton base or fiber mutations, or penton interserotypic chimeras. EM observations of cells infected with Ad5 carrying knob-ablated fibers (Ad5R7Δknob) confirmed that the knob domain was involved in crystal genesis and molecular structure. In the absence of knob, the protein crystals were different from those observed in WT Ad5-infected cells, in that they were smaller and arranged as macles (Fig. 5 A). They still reacted with penton base antibody, confirming that penton base was the other protein component of the crystals (Fig. 5 C), and their parameters were compatible with a tubular unit formed of penton base capsomers prolonged with knobless fibers (Fig. 13). Ad5 penton base mutant Ad5PbEGD induced crystals with WT characteristics, excluding the RGD motifs from a role in the crystal structure.

The fiber shaft domain was also involved in crystal genesis and structure, as shown by the morphology of crystalline inclusions induced by the shaft deletion mutant Ad5ΔR4-R16, lacking shaft repeats 4 to 16 (Fig. 8 A, B), and the absence of crystals in Ad5ΔR4-R19- and Ad5ΔR8-R21-infected cells. The involvement of the shaft domain was further analyzed using Ad5 with penton chimeras. No crystals were observed in cells infected with Ad5/K3, carrying the Ad3 knob domain at the extremity of an Ad5 fiber shaft. However, when knob and shaft were homotypic, e.g. in Ad5/F3, small protein crystals were observed (Fig. 8 C–E). Likewise Ad5/F35, a chimeric vector pseudotyped with serotype 5 fiber, formed intranuclear crystals, although with discontinuities in the tubular lattice (Fig. 9).

Further mapping of the knob region(s) implicated in crystal formation or/and structure was performed using substitutions, small insertions or deletions in the knob domain. The occurrence of crystals in Ad5ΔEF-infected cells (Fig. 6 A–C), compared to the absence of crystals with mutant Ad5ΔTAYT489 (Fig. 6 D), indicated that the short double strand EF was dispensable for crystal formation, but that the region immediately downstream in the FG loop, spanning residues 489–492, was crucial for crystallogenesis (Fig. 4 B). It is noteworthy that this region coincides with part of the CAR-binding domain in the knob [31]. The two main regions of the shaft and knob domains of Ad5 fiber which were identified as essential for crystal formation are schematically represented in Fig. 4 (shaded vertical bars).

The irregular and wavy aspect of crystals induced by the substitution mutant Ad5F497D might let suppose that the F residue of the highly conserved motif FMP motif at position 497–499 in Ad5 fiber was a major determinant of the crystal structure (Fig. 7 A, B). However, the barely detectable level of fiber protein expression in Ad5F497D-infected cells (Fig. 7 C) indicated that the crytal-like inclusions in these cells were constituted of penton base protein alone, and suggested that the lack of fiber projection was responsible for the structural irregularity of the crystal lattice.

### Structural model of intranuclear penton crystal

Image processing of the tubular unit of chimeric Ad5/F3 penton crystal seen in cross-section as in Fig. 8 D, showed striking similarity in dimensions and shape with the 2D projection of Ad3 dodecahedron, i.e. penton dodecamer with its 12 short fibers (Fig. 13 A, B). We therefore propose a model for the Ad5 penton crystalline inclusions, in which the penton capsomers would associate into dodecahedrons disposed in parallel rows (Fig. 13 C–E). Although this model is based on the Ad3 dodecahedron well-characterized structure, it has been shown that penton base of Ad2, which has only seven amino acid differences with that of Ad5 [50], can form dodecahedrons upon certain solvent conditions affecting the dodecamer-to-pentamer equilibrium [51]. Likewise, recombinant Ad2 penton base mutant W119H, has been found to spontaneously assemble into dodecahedrons instead of accumulating in the pentameric form [52]. However, we cannot exclude another crystal model in which pentons would be arranged in parallel tubular structures: cryotomographic analysis of crystals might help resolving this ambiguity.

### Biological function(s) of nuclear protein crystals in the Ad life cycle: byproducts, storage or virus assembly platform?

#### Theoretical considerations

The fiber protein in Ad2- or Ad5-infected cells is in large excess compared to penton base, which represents the limiting factor in the penton capsomer assembly reaction in vivo [39]–[41] (and refer to Fig. 7 C). The reaction of binding of fiber to penton base is the only virus assembly step which has been reproduced in vitro so far from two separate capsid elements, and the dissociation constant of the in vitro assembly reaction of Ad2 WT fiber with penton base has been estimated to be *K*_d_ = 2×10^−7^ M in terms of fiber molarity [53]. In this context, one might legitimately raise the question of the reason(s) or/and selective advantage for the virus to accumulate penton crystalline inclusions within the nucleoplasm of the infected cell. A clue to the role of penton crystals in the Ad life cycle was provided by Ad mutants defective in virus assembly.

#### Observations

If the intranuclear penton crystals represented byproducts of viral capsid components synthesized in vast excess in Ad-infected cells, Ad mutants or recombinants defective in virus assembly should accumulate crystals in greater abundancy than in WT Ad-infected cells, assuming equivalent levels of virus protein synthesis. Such a pattern was never observed [54]. In the same line of data, Ad recombinants with a low level of fiber encapsidation efficiency and/or low infectivity failed to produce regular crystalline inclusions of penton protein in 293 cells. This was the case for Ad5ΔR4-R19 and Ad5R7Δknob, as well as for Ad5F497D [19]. This correlation between the lack of intranuclear penton crystals and defect in virus assembly and viability argued against the crystals being simple byproducts from virus protein synthesis. This was confirmed by the kinetics of appearance of crystals within the nucleus of Ad-infected cells: intranuclear crystals were visible as early as 16 h pi, i.e. at early steps of the late phase of the virus life cycle (Fig. 3 I), indicating that crystal formation was contemporary to virion assembly.

This was also confirmed by our double fiber-expressing vector Ad5/R7-ZZ_wt_/E1:WTFib. We have shown that the deletion of the knob had a negative effect on the rate of translation of the knob-deleted, short-shafted and Z_wt_-liganded fiber protein R7-ZZ_wt_ in Ad5/R7-ZZ_wt_-infected 293 cells. The same effect was observed in cells infected with Ad5/R7-ZZ_wt_/E1:WTFib, a recombinant with two fiber genes, one coding for WT fiber, the other for R7-ZZ_wt_ [18]. Both WT and R7-ZZ_wt_ fiber protein species had the same tail domain which contained the penton base binding site and trimerized with the same apparent efficiency, but R7-ZZ_wt_ fiber showed a lower efficiency of encapsidation [18]. Although it could not be excluded some difference in the affinity for penton base between WT and R7-ZZ_wt_ fibers, the most likely explanation would be that the compartmentalization of penton protein would be the major cellular determinant of its packaging into virions: localization of WT penton in intranuclear crystals would favor their usage as building blocks for Ad capsids, as suggested by the pattern of virus particle assembly in the nucleus of Ad5/R7-ZZ_wt_/E1:WTFib-infected cells (Fig. 10), as well as the fiber composition of the viral progeny (Fig. 11 F).

#### Hypothesis

The temporal and spatial relations between penton crystals and Ad virions, e.g. the kinetics of crystal occurrence (Fig. 3 H–J), and the intimate contacts between intranuclear virions and crystals in Ad5WTFib-infected cells (Fig. 1 and 2), as well as the arguments developed above strongly suggested that penton crystalline inclusions played a role in the Ad morphogenic process. We hypothesize that penton crystals represent a privileged assembly platform providing the virus assembly machinery with a large supply of preassembled penton capsomers, the major limiting factor in capsid assembly [39]–[41]. Alternatively, the high concentration of penton capsomers in crystals would constitute a nucleation centre for the Ad assembly machinery. This does not preclude the existence of other but less efficient assembly sites within the nucleoplasm.

### Crystal inclusions as a marker of Ad assembly efficiency and viability

Our mAb TB5, which was originally raised against Ad5 fiber and cross-reacted with Ad2 and Ad3 fibers, was found to react with high efficiency with penton protein crystals *in situ* in IF microscopy. This implied that the TB5 epitope was accessible within the intranuclear crystalline inclusions, and not buried in contacts between crystal components. However, precise mapping of the TB5 epitope required further experiments, and only indirect conclusions could be drawn from our present data. The absence of reaction of TB5 with Ad5/F35, compared to its positive reaction with Ad5/F3 and Ad5/F5, suggested that the TB5 epitope corresponded to a region of the fiber knob which was in common with two serotypes belonging to two different subgroups, i.e. C-Ad5 and B-Ad3, but differed between two serotypes of the same subgroup, i.e. B-Ad3 [34] and B-Ad35 [55]. Ad5ΔEF-, Ad5V441G-, Ad5K442A-, Ad5H508A- and Ad5(HI)RGD4C-infected cells reacted with TB5 with a similar intensity, and revealed the same rod-like intranuclear inclusions as WT (Fig. 3 A–D and Fig. 6 B, inset), which excluded the regions within residues 441–442, 479–486, histidine-508, and the HI loop as directly involved in TB5 binding. Likewise, region 489–492 could be excluded since amorphous nuclear inclusions reacted with TB5 in Ad5ΔTAYT489-infected cells (Fig. 6 D, inset). This indicated that the TB5 epitope could also react with fiber protein in other contexts than that of penton crystals. All the above cited residues, except for the HI loop, were involved in CAR recognition [31], which implied that the TB5 epitope did not belong to the CAR-binding surface of the fiber knob.

As the absence of protein inclusions with regular structure in Ad-infected cells correlated with a low fiber content and low infectivity of virus progeny, Ad-induced intranuclear protein inclusions could serve as a good criterion of proper capsid assembly, fiber expression level and encapsidation efficiency, and virus infectivity. Thus, we propose to use the IF pattern of Ad-infected 293 cells with mAb TB5 as a simple and rapid prognostic assay for the viability and productivity of fiber-modified Ad vectors.

## Methods

### Cell culture

E1A-E1B-*trans*-complementing HEK-293 cell line (abbreviated 293; CRL 1573) and insect cell line Sf9 (CRL 1711) were obtained from the American Type Culture Collection (Manassas, Va). The 293-derived fiber-*trans*-complementing cell line, abbreviated 293-Fiber, was obtained from Transgene SA (Strasbourg, France). Mammalian cells were cultured as monolayers in DMEM supplemented with 10% fetal calf serum (FCS, Sigma), penicillin (200 U/mL), and streptomycin (200 µg/mL; Gibco-Invitrogen) at 37°C and 5% CO_2_. For growth of 293-Fiber cells, hygromycin was added at 350 µg/mL [6]. Insect Sf9 cells were grown as monolayers in Grace's medium supplemented with 10% FCS, as described elsewhere [52].

### Construction and nomenclature of recombinant Ad with modified fiber and penton base

The construction of fiber- or penton base-modified Ad has been described in detail in previous studies [15]–[17], [56], [57]. The choice of acronyms for the recombinant Ad used in the present study was determined by their penton base or fiber modification (Fig. 1), regardless of their reporter gene, coding for GFP or beta-galactosidase (ßGal). ***(i) Penton base mutant***. The penton base mutant Ad5Pb-EGD, encoding GFP and carrying a RGD-to-EGD substitution at position 340 in the penton base coding sequence, has been described elsewhere [57]. All the other recombinant Ads carried modifications in their fiber genes. ***(ii) Fiber shaft deletion mutants***. Ad5ΔR4-R16 and Ad5ΔR4-R19, which coded for ßGal, had a shorter fiber shaft domain resulting from the deletion of repeats 4 to 16 and 4 to 19, respectively [58]. Ad5ΔR8-R21 coded for GFP and was called Ad5/R7-knob in previous studies [15]–[17], [56]. The deletion in Ad5ΔR8-R21 overlapped the shaft repeats 8 to 21, and resulted from the junction of Ala158, the second residue of repeat 8, to the first residue (Ile389) of repeat 22. Ad5ΔR8-R21 also carried an insertion of an extrinsic trimerisation signal corresponding to the neck region peptide (NRP) from the human lung surfactant protein D. ***(iii) Fiber knob deletion mutants***. Ad5R7ΔKnob, carrying a short shafted fiber with seven repeats and complete deletion of the knob, has been described in detail in previous studies [15]–[17], [56]. Ad5ΔEF, had the eight amino acid residues 479–486 deleted, resulting in the absence of the short double beta-strand EF [32], [33]. Ad5ΔTAYT489, which carried a four amino acid deletion (489-TAYT-492), has been previously charaterized [59]. ***(iv) Fiber knob substitution mutants***. Fiber substitutions V441G, K442A and H508A were introduced in shuttle plasmids using standard molecular biology techniques. Plasmid adenoviral backbones were further obtained by homologous recombination in *E. coli* [60] with AE18, a LacZ-encoding Ad5 backbone [59]. All plasmids were checked by restriction analysis and DNA sequencing. Mutant Ad5F497D has been described elsewhere [19]. ***(v) Fiber knob insertion mutant.*** Ad5(HI)RGD4C was a ßGal-expressing vector carrying the insertion of a cyclic RGD motif (CDCRGDCFC, abbreviated RGD4C) in the HI loop of the knob domain [57], [61]. ***(vi) Chimeric pentons***. Ad5ßGal vectors carrying Ad5/Ad3 chimeric fibers consisted of Ad5/K3, in which only the knob domain of serotype 3 fiber was fused to the last repeat of Ad5 shaft, and Ad5/F3, in which the serotype 3 shaft and knob domains were fused to the Ad5 fiber tail [58]. Ad5/F35 was a GFP-expressing vector carrying the shaft and knob domains of serotype 35 fiber fused to Ad5 fiber tail, and reinserted into the Ad5 genome in place of the Ad5 fiber gene. A schematic representation of these recombinant Ads is shown in Fig. 4.

### Construction of recombinant Ad5 carrying two fiber genes

The genome of Ad5/R7-ZZ_wt_/E1:WTFib contained two genes coding for two different fiber proteins, WT-fiber and R7-ZZ_wt_ fiber. R7-ZZ_wt_ was a short-shafted (R7, seven repeats), knob-deleted fiber carrying the trimerisation motif from the neck region peptide (NRP) of the human lung surfactant protein D [18] and a tandem Z_wt_-ligand (ZZ_wt_). Z_wt_ corresponded to an Ig-binding domain derived from the *Staphylococcal* protein A, and has been described in previous studies [18], [37], [38]. The gene encoding the R7-ZZ_wt_ fiber was at its normal location in the L5 region of the Ad5/R7-ZZ_wt_/E1:WTFib genome, whereas an ectopic WT-fiber gene was inserted into the deleted E1 region [18]. Ad5/R7-ZZ_wt_/E1:WTFib was constructed as follows. The gene for WT-fiber was inserted into the E1 region of the cloning plasmid pAdTrack-CMV [62] and recombined with pAdEasy-1. This resulted in an Ad5 genome in which the E1 region was replaced by the WT Ad5 fiber gene driven by the immediate-early CMV promoter, and the GFP gene driven by another CMV promoter. This genome with two WT fiber genes was designated Ad5/WT-fiber/E1:WT-fiber. Ad5/WT-fiber/E1:WT-fiber was then restricted with *Pac* I/*Spe* I, and ligated to a genome with a truncated fiber gene containing the coding sequence for a tandem Z_wt_ ligand (R7-ZZ_wt_ fiber), as described previously [15], thus generating the recombinant genome Ad5/R7-ZZ_wt_/E1:WTFib.

### Insect cells and recombinant baculoviruses

*Spodoptera frugiperda* cells, Sf9 subclone, were maintained as monolayers in Grace's medium supplemented with 10% fetal bovine serum, and infected with recombinant baculoviruses at a multiplicity of infection (MOI) ranging from 5 to 10 PFU/cell. The construction of recombinant baculoviruses derived from *Autographa californica* MultiCapsid NucleoPolyhedrosis Virus (AcMNPV) and expressing Ad2 fiber, Ad2, Ad5 or Ad3 fiber knob, or Ad2 penton base under the control of the polyhedrin promoter has been described in detail in previous studies [49], [52].

### Antibodies and immunological assays for fiber and penton base

MAb anti-fiber 4D2.5 and 2A6.36 [63] were obtained from Jeff Engler (University of Alabama at Birmingham). Polyclonal antibody against Ad fiber (laboratory-made) was raised in rabbit by injection of a mixture of chromatographically purified native and SDS-denatured recombinant Ad2 fiber protein [4], [64], [65], and polyclonal antibody against Ad penton base (laboratory-made) was raised in rabbit by injection of chromatograpically purified recombinant Ad2 penton base [49], [52]. Mouse monoclonal antibodies against hexon group-specific epitopes were purchased from Chemicon Intl. (Temecula, CA) for MAB8051 and MAB8043, and obtained from W.C. Russell (St Andrews University, Scotland) for 4C3 [66]. Mouse polyclonal anti-pIIIa, anti-core protein V and anti-core protein VII antibodies were raised in mice by injection of the desired proteins excised from Coomassie blue-stained gels of CsCl gradient-purified, SDS-denatured Ad5 virions. The specificity of each antibody was verified in Western blot analysis of SDS-denatured Ad5 virions (data not shown).

The mouse monoclonal anti-fiber antibody TB5 was generated by immunisation of a Balb/C mouse with whole cell extracts of Balb/C 3T3 cells infected with Ad5. In brief, 10^8^ Ad5-infected 3T3 cells were suspended in 0.5 ml PBS, sonicated in a probe sonicator, emulsified with Complete Freund's adjuvant (CFA) and injected intra-dermally and intra-peritoneally into a 10 week-old Balb/C mouse, followed by a boost of 2×10^7^ Ad5-infected 3T3 cells in CFA 10 days later. After a two week interval, the mouse was given an intra-peritoneal injection of sonicated Ad5-infected 3T3 cells (3×10^7^ cells) in PBS and four days later, an identical intravenous boost, followed by sacrifice three days later. Cell fusion was performed using mouse splenocytes and NSO mouse myeloma cells using standard techniques [67]. Hybridoma culture supernatants were screened by immunoprecipitation of extracts of (^35^S)-methionine-labeled Ad5-infected Hep2 cells according to the method of Cepko et al [68]. One hybridoma cell culture (termed TB5) was selected on the basis of specific immunoprecipitation of the fiber protein by secreted antibody. The TB5 hybridoma cells were cloned in soft agar, culture supernatant was harvested and used in immunocytochemistry.

Fiber and penton base proteins were assayed in cell lysates or in CsCl gradient-purified virion samples using SDS-PAGE and quantitative Western blot analysis. Blots were incubated with 4D2.5 or anti-penton base primary antibodies followed by secondary (^35^S)LR-labeled anti-mouse or anti-rabbit whole IgG antibody (GE Healthcare Bio-Sciences; 2,000 Ci/mmol; 20–30 µCi per 100 cm^2^ membrane), and subjected to autoradiography (Hyperfilm™ MP, GE Heathcare Bio-Sciences). Protein bands were excised from blots and radioactivity measured in a scintillation counter (Beckman LS-6500), as previously described [69]. Alternatively, autoradiographs were scanned and quantitated by densitometric analysis, using the VersaDoc image analyzer and the Quantity One program (BioRad).

### Immunofluorescence (IF) microscopy

Cell monolayers were harvested at 48 h post-infection, fixed with 2% paraformaldehyde in phosphate buffered saline (PBS) and permeabilized in 0.2% (v/v) Triton X-100 in PBS. Cells were blocked with 1% BSA in PBS (PBS-BSA), and reacted with rabbit anti-penton base or anti-fiber antibody (1∶200 in PBS-BSA) and Alexa Fluor® 488-labeled goat anti-rabbit IgG (Molecular Probes, Invitrogen), or mAb anti-fiber TB5 (1∶200 in PBS-BSA) and Alexa Fluor® 633-labeled goat anti-mouse IgG antibody (Molecular Probes, Invitrogen). Samples were treated with DAPI and mounted on slides. For conventional fluorescence microscopy, images were acquired using an Axiovert 135 inverted microscope (Zeiss) equiped with an AxioCam video camera. For confocal microscopy, samples were analyzed using a Leica TCS SP2 confocal microscope.

### Electron microscopy (EM), immuno-electron microscopy (Immuno-EM) and image processing

***(i) EM analysis of cell sections***. Cells were harvested at 48 h after infection, pelleted, fixed with 2% glutaraldehyde in 0.1 M sodium cacodylate buffer, pH 7.4, and post-fixed with osmium tetroxide (1% in 0.1 M cacodylate buffer, pH 7.4). Cell specimens were dehydrated and embedded in Epon (Epon-812; Fulham, Latham, NY). Sections were stained with 7% uranyl acetate in methanol, post-stained with 2.6% alkaline lead citrate in H_2_O, and examined under a Jeol JEM-1400 electron microscope, equipped with an ORIUS™ digital camera (Gatan France, 78113-Grandchamp). Measurements of crystal parameters were made using the camera imaging software. ***(ii) Immuno-EM analysis of cell sections*** Cell specimens were included in metacrylate resin (LR White Resin, London Resin Company, Reading, UK). Sections on grids were first quenched for non-specific antibody binding as previously described [70], [71], then reacted with primary rabbit anti-fiber or anti-penton base antibody used at a dilution of 1∶50 to 1∶500 in Tris-buffered saline (TBS) containing 1% bovine serum albumin (TBS-BSA), and incubated overnight at 4°C. After rinsing in TBS-BSA and H_2_O, sections were post-incubated with 10-nm colloidal gold-conjugated goat anti-rabbit IgG antibody (British BioCell International, Cardiff, UK) diluted to 1∶50 in TBS for 1 h at room temperature. Alternatively, 5-nm colloidal gold-labeled protein A (British BioCell International) was used to detect the primary antibody. MAb TB5 was used as undiluted supernatant of hybridoma cell culture, and detected using 10-nm colloidal gold-conjugated rabbit anti-mouse IgG antibody diluted as above. Controls consisted of specimens on grids treated in the same manner, except that primary antibodies were omitted. Specimens were contrasted with 2% uranyl acetate in H_2_O, and examined under the Jeol JEM-1400 electron microscope, as above. ***(iii)*** ** ***Negative staining of Ad particles***. Samples were applied to the clean side of carbon on mica (carbon/mica interface) and negatively stained with 1% ammonium molybdate, pH 7.5. A grid was placed on top of the carbon film, and subsequently air-dried. Micrographs were taken under low-dose conditions with a Jeol 1200-EX II microscope at 100 kV and a calibrated magnification of 39,750 times (based on the helical pitch of Tobacco Mosaic Virus). Selected negatives were digitalized on a Zeiss scanner (Photoscan TD) with a pixel size of 14 µm, corresponding to 3.5 Å at the sample scale, as given by the following calculation: 140,000 Å (scanning step size)/39,750 (microscope magnification) = 3.52 Å. ***(iv)*** ** ***Immunogold staining of Ad particles***. R7-ZZ_wt_ fibers carried by Ad particles were labeled as follows. A 4 µl-sample of virus suspension was deposited on top of a carbon-coated grid. 30 sec later, the excess of liquid was removed by blotting with filter paper. 4 µl of a 100-fold diluted solution of 6-nm colloidal gold-labeled antibody (6-nm AffiniPure goat anti-human IgG, EM grade; Jackson ImmunoResearch) was placed on the grid and incubated for 1 min at room temperature. The antibody solution was then removed by filter paper adsorption, and replaced by 4 µl of stain (2% ammonium molybdate, pH 7.4). After a further 30 sec, the grid was dried on filter paper, and examined under the electron microscope as above. ***(v)*** ***Image processing and model representation***. 2D averaging of intranuclear Ad5/F3 protein crystal was performed as follows. A total of 40 overlapping fields coming from a single crystal (as shown in Fig. 8 D) were cut out using the X3d program [72], and averaged after cross-correlation. The cryoelectron microscopic (cryoEM) structure of the Ad3 dodecahedron with 12 fiber projections (dodecahedron-fibers) was downloaded from the EM database associated with the Macromolecular Structure Database (http://www.ebi.ac.uk/msd-srv/emsearch/index.html). The 3D map of Ad3 dodecahedron-fibers was filtered to 45Å and reprojected along its 3-fold axis using the SPIDER program [73]. 3D isosurface representation was visualized using WEB [73].

## References

[1] Russell WC (2000). Update on adenovirus and its vectors.. J Gen Virol.

[2] Hong JS, Engler JA (1996). Domains required for assembly of adenovirus type 2 fiber trimers.. J Virol.

[3] Li J, Lad S, Yang G, Luo Y, Iacobelli-Martinez M (2006). Adenovirus fiber shaft contains a trimerization element that supports peptide fusion for targeted gene delivery.. J Virol.

[4] Novelli A, Boulanger P (1991). Deletion analysis of functional domains in baculovirus-expressed adenovirus type 2 fiber.. Virology.

[5] Novelli A, Boulanger PA (1991). Assembly of adenovirus type 2 fiber synthesized in cell-free translation system.. J Biol Chem.

[6] Gaden F, Franqueville L, Magnusson MK, Hong SS, Merten MD (2004). Gene transduction and cell entry pathway of fiber-modified Adenovirus type 5 vectors carrying novel endocytic peptide ligands selected on human tracheal glandular cells.. J Virol.

[7] Legrand V, Spehner D, Schlesinger Y, Settelen N, Pavirani A (1999). Fiberless recombinant adenoviruses: virus maturation and infectivity in the absence of fiber.. J Virol.

[8] Miyazawa N, Leopold PL, Hackett NR, Ferris B, Worgall S (1999). Fiber swap between adenovirus subgroups B and C alters intracellular trafficking of adenovirus gene transfer vectors.. J Virol.

[9] Miyazawa N, Crystal RG, Leopold PL (2001). Adenovirus serotype 7 retention in a late endosomal compartment prior to cytosol escape is modulated by fiber protein.. J Virol.

[10] Shayakhmetov DM, Li ZY, Ternovoi V, Gaggar A, Gharwan H (2003). The interaction between the fiber knob domain and the cellular attachment receptor determines the intracellular trafficking route of adenoviruses.. J Virol.

[11] Walters RW, Freimuth P, Moninger TO, Ganske I, Zabner J (2002). Adenovirus fiber disrupts CAR-mediated intercellular adhesion allowing virus escape.. Cell.

[12] Shayakhmetov DM, Lieber A (2000). Dependence of adenovirus infectivity on length of the fiber shaft domain.. J Virol.

[13] Wu E, Pache L, Von Seggern DJ, Mullen T-M, Mikyas Y (2003). Flexibility of the adenovirus fiber is required for efficient receptor interaction.. J Virol.

[14] Bayo-Puxan N, Castello M, Gros A, Huch M, Fillat C (2006). Role of the putative heparan sulfate glycosaminoglycan-binding site of the adenovirus type 5 fibre shaft on liver detargeting and knob-mediated retargeting.. J Gen Virol.

[15] Magnusson MK, Hong SS, Boulanger P, Lindholm L (2001). Genetic retargeting of adenovirus: novel strategy employing “deknobbing” of the fiber.. J Virol.

[16] Magnusson MK, Hong SS, Henning P, Boulanger P, Lindholm L (2002). Genetic retargeting of adenovirus vectors: functionality of targeting ligands and their influence on virus viability.. J Gene Med.

[17] Henning P, Magnusson MK, Gunneriusson E, Hong SS, Boulanger P (2002). Genetic modification of adenovirus 5 tropism by a novel class of ligands based on a three-helix bundle scaffold derived from staphylococcal protein A.. Hum Gene Ther.

[18] Henning P, Lundgren E, Carlsson M, Frykholm K, Johannisson J (2006). Adenovirus type 5 fiber knob domain has a critical role in fiber protein synthesis and encapsidation.. J Gen Virol.

[19] Leissner P, Legrand V, Schlesinger Y, Hadji DA, van Raaij M (2001). Influence of adenoviral fiber mutations on viral encapsidation, infectivity and in vivo tropism.. Gene Ther.

[20] Von Seggern DJ, Chiu CY, Fleck SK, Stewart PL, Nemerow GR (1999). A helper-independent adenovirus vector with E1, E3, and fiber deleted: structure and infectivity of fiberless particles.. J Virol.

[21] Boulanger P, Torpier G, Biserte G (1970). Investigation on intranuclear paracrystalline inclusions induced by adenovirus 5 in KB cells.. J Gen Virol.

[22] Boulanger PA, Torpier G, Rimsky A (1974). Crystallographic study of intranuclear adenovirus type 5 crystals.. Intervirology.

[23] Henry CJ, Atchison RW (1971). Paracrystalline formation in cell cultures infected with adenovirus type 2.. J Virol.

[24] Henry CJ, Slifkin M, Merkow LP, Pardo M (1971). The ultrastructure and nature of adenovirus type 2-induced paracrystalline formations.. Virology.

[25] Lifchitz A, Rimsky A, Torpier G, Boulanger P (1975). Crystallographic analysis of an adenovirus-induced protein crystal in KB cell: a structural model.. Acta Cryst B.

[26] Marusyk R, Norrby E, Marusyk H (1972). The relationship of adenovirus-induced paracrystalline structures to the virus core protein(s).. J Gen Virol.

[27] Weber J, Liao S-K (1969). Light and electron microscopy of virus-associated intranuclear paracrystals in cultured cells infected with type 2, 4, 6 and 18 human adenoviruses.. Can J Microbiol.

[28] Wills EJ, Russell WC, Williams JF (1973). Adenovirus-induced crystals : studies with temperature-sensitive mutants.. J Gen Virol.

[29] Hong SS, Boulanger P (1995). Protein ligands of the human adenovirus type 2 outer capsid identified by biopanning of a phage-displayed peptide library on separate domains of wild-type and mutant penton capsomers.. EMBO J.

[30] Morgan C, Godman GD, Breitenfield PM, Rose HM (1960). A correlative study by electron and light microscopy of the development of type 5 adenovirus. I. Electron microscopy.. J Exp Med.

[31] Law LK, Davidson BL (2005). What does it take to bind CAR?. Mol Ther.

[32] Santis G, Legrand V, Hong SS, Davison E, Kirby I (1999). Molecular determinants of adenovirus serotype 5 fibre binding to its cellular receptor CAR.. J Gen Virol.

[33] Kirby I, Davison E, Beavil AJ, Soh CP, Wickham TJ (1999). Mutations in the DG loop of adenovirus type 5 fiber knob protein abolish high-affinity binding to its cellular receptor CAR.. J Virol.

[34] Chroboczek J, Ruigrok RW, Cusack S, Doerfler W, Böhm P (1995). Adenovirus fiber.. The molecular repertoire of adenoviruses I Virion structure and infection Curr Top Microbiol Immunol, pp163–200.

[35] Cauet G, Strub JM, Leize E, Wagner E, Van Dorsselaer A (2005). Identification of the glycosylation site of the adenovirus type 5 fiber protein.. Biochemistry.

[36] Ruigrok RW, Barge A, Albiges-Rizo C, Dayan S (1990). Structure of adenovirus fibre. II. Morphology of single fibres.. J Mol Biol.

[37] Henning P, Andersson KME, Frykholm K, Ali A, Magnusson MK (2005). Tumor cell targeted gene delivery by adenovirus 5 vectors carrying knobless fibers with antibody-binding domains.. Gene Ther.

[38] Magnusson MK, Henning P, Myhre S, Wikman M, Uil TG (2007). Adenovirus 5 vector genetically re-targeted by an Affibody molecule with specificity for tumor antigen HER2/neu.. Cancer Gene Ther.

[39] Boudin M-L, Moncany M, D'Halluin JC, Boulanger PA (1979). Isolation and characterization of adenovirus type 2 vertex capsomer (penton base).. Virology.

[40] Boudin M-L, Rigolet M, Lemay P, Galibert F, Boulanger P (1983). Biochemical and genetical characterization of a fiber-defective temperature-sensitive mutant of type 2 adenovirus.. EMBO J.

[41] D'Halluin J-C, Doerfler W, Böhm P (1995). Adenovirus assembly.. The molecular repertoire of adenoviruses I Virion structure and infection Curr Top Microbiol Immunol, pp 47–66.

[42] Nilsson B, Moks T, Jansson B, Abrahmsén L, Elmblad A (1987). A synthetic IgG-binding domain based on staphylococcal protein A.. Protein Eng.

[43] Puvion-Dutilleul F, Legrand V, Mehtali M, Chelbi-Alix MK, de The H (1999). Deletion of the fiber gene induces the storage of hexon and penton base proteins in PML/Sp100-containing inclusions during adenovirus infection.. Biol Cell.

[44] Souquere-Besse S, Pichard E, Fihol O, Legrand V, Rosa-Calatrava M (2002). Adenovirus infection targets the cellular protein kinase CK2 and RNA-activated protein kinase (PKR) into viral inclusions of the cell nucleus.. Micros Res & Technique.

[45] Weber J (1978). Physical mapping of the genes controlling adenovirus paracrystal formation.. Can J Microbiol.

[46] Horwitz MS, Fields BN, Knipe DM (1986). Adenoviruses and their replication.. Fundamental virology.

[47] Coulibaly F, Chiu E, Ikeda K, Gutmann S, Haebel PW (2007). The molecular organization of cypovirus polyhedra.. Nature.

[48] Rey FA (2007). Virology: holed up in a natural crystal.. Nature.

[49] Karayan L, Gay B, Gerfaux J, Boulanger P (1994). Oligomerization of recombinant penton base of adenovirus type 2 and its assembly with fiber in baculovirus-infected cells.. Virology.

[50] Neumann R, Chroboczek J, Jacrot B (1988). Determination of the nucleotide sequence for the penton base gene of human adenovirus type 5.. Gene.

[51] Zubieta C, Blanchoin L, Cusack S (2006). Structural and biochemical characterization of a human adenovirus 2/12 penton base chimera.. FEBS J.

[52] Karayan L, Hong SS, Gay B, Tournier J, d'Angeac AD (1997). Structural and functional determinants in adenovirus type 2 penton base recombinant protein.. J Virol.

[53] Boudin M-L, Boulanger PA (1982). Assembly of adenovirus penton base and fiber.. Virology.

[54] Martin GR, Warocquier R, Cousin C, D'Halluin J-C, Boulanger PA (1978). Isolation and phenotypic characterization of human adenovirus type 2 temperature-sensitive mutants.. J Gen Virol.

[55] Kovacs GM, Davison AJ, Zakhartchouk AN, Harrach B (2004). Analysis of the first complete genome sequence of an Old World monkey adenovirus reveals a lineage distinct fom the six human adenovirus species.. J Gen Virol.

[56] Hong SS, Magnusson MK, Henning P, Lindholm L, Boulanger PA (2003). Adenovirus stripping : a versatile method to generate adenovirus vectors with new cell target specificity.. Mol Ther.

[57] Waszak P, Franqueville L, Franco-Motoya M-L, Rosa-Calatrava M, Boucherat O (2007). Toxicity of fiber- and penton base-modified adenovirus type 5 vectors on lung development in newborn rats.. Mol Ther.

[58] Vigne E, Dedieu JF, Brie A, Gillardeaux A, Briot D (2003). Genetic manipulations of adenovirus type 5 fiber resulting in liver tropism attenuation.. Gene Ther.

[59] Martin K, Brie A, Saulnier P, Perricaudet M, Yeh P (2003). Simultaneous CAR- and alpha V integrin-binding ablation fails to reduce Ad5 liver tropism.. Mol Ther.

[60] Crouzet J, Naudin L, Orsini C, Vigne E, Ferrero L (1997). Recombinational construction in Escherichia coli of infectious adenoviral genomes.. Proc Natl Acad Sci USA.

[61] Dmitriev I, Krasnykh V, Miller CR, Wang M, Kashentseva E (1998). An adenovirus vector with genetically modified fibers demonstrates expanded tropism via utilization of a coxsackievirus and adenovirus receptor-independent cell entry mechanism.. J Virol.

[62] He TC, Zhou S, da Costa LT, Yu J, Kinzler KW (1998). A simplified system for generating recombinant adenoviruses.. Proc Natl Acad Sci U S A.

[63] Hong JS, Engler JA (1991). The amino terminus of the adenovirus fiber protein encodes the nuclear localization signal.. Virology.

[64] Boulanger PA, Puvion F (1973). Large-scale preparation of soluble adenovirus hexon, penton and fiber antigens in highly purified form.. Eur J Biochem.

[65] Molinier-Frenkel V, Lengagne R, Gaden F, Hong SS, Choppin J (2002). Adenovirus hexon protein is a potent adjuvant for activation of a cellular immune response.. J Virol.

[66] Hong SS, Szolajska E, Schoehn G, Franqueville L, Myhre S (2005). The 100K-chaperone protein from adenovirus serotype 2 (Subgroup C) assists in trimerization and nuclear localization of hexons from subgroups C and B adenoviruses.. J Mol Biol.

[67] Galfre G, Milstein C (1981). Preparation of monoclonal antibodies : strategies and procedures.. Methods Enzymol.

[68] Cepko CL, Sharp PA (1982). Assembly of adenovirus major capsid protein is mediated by a nonvirion protein.. Cell.

[69] Huvent I, Hong SS, Fournier C, Gay B, Tournier J (1998). Interaction and co-encapsidation of HIV-1 Vif and Gag recombinant proteins.. J Gen Virol.

[70] Hong SS, Gay B, Karayan L, Dabauvalle MC, Boulanger P (1999). Cellular uptake and nuclear delivery of recombinant adenovirus penton base.. Virology.

[71] Violot S, Hong SS, Rakotobe D, Petit C, Gay B (2003). The human Polycomb-group EED protein interacts with the integrase of human immunodeficiency virus type 1 (HIV-1).. J Virol.

[72] Conway JF, Steven AC (1999). Methods for reconstructing density maps of ‘single’ particles from cryoelectron microfraphs to subnanometer resolution.. J Struct Biol.

[73] Frank J, Radermacher M, Penczek P, Zhu J, Li Y (1996). SPIDER and WEB : processing and visualization of images in 3D electron microscopy and related fields.. J Struct Biol.

